# Coarse extrinsic curvature of Riemannian submanifolds

**DOI:** 10.1007/s40879-025-00816-x

**Published:** 2025-04-10

**Authors:** Marc Arnaudon, Xue-Mei Li, Benedikt Petko

**Affiliations:** 1 CNRS, Bordeaux INP, IMB, UMR 5251, University Bordeaux, 33400 Talence, France; 2 Department of Mathematics, Imperial College London, London, UK; 3 EPFL, Lausanne, Switzerland

**Keywords:** Coarse curvature, Extrinsic curvature, Optimal transport, 53B25, 49Q22

## Abstract

We introduce a novel concept of coarse extrinsic curvature for Riemannian submanifolds, inspired by Ollivier’s notion of coarse Ricci curvature. This curvature is derived from the Wasserstein 1-distance between probability measures supported in the tubular neighborhood of a submanifold, providing new insights into the extrinsic curvature of isometrically embedded manifolds in Euclidean spaces. The framework also offers a method to approximate the mean curvature from statistical data, such as point clouds generated by a Poisson point process. This approach has potential applications in manifold learning and the study of metric embeddings, enabling the inference of geometric information from empirical data.

## Introduction

Synthetic lower bounds on Ricci curvature is a powerful tool in the study of classical geometric analysis and metric measure spaces. Ollivier’s notion of Coarse Ricci Curvature is distinct in that it approximates the curvature itself, rather than merely providing a lower bound. By selecting as test measures weighted localized volume measures, supported on a ball of radius $\varepsilon$, the Wasserstein 1-distance between two such measures reveals the generalized Ricci tensor; applying to random geometric graphs sampled from a Poisson point process with non-uniform intensity leads to similar conclusions [2].

Inspired by the concept of coarse Ricci curvature, in this article we seek a suitable notion of extrinsic curvature for embedded manifolds. With manifold learning applications in mind, we initially work with curves and surfaces and subsequently define a concept of coarse extrinsic curvature for general embedded manifolds. This notion captures the inner product between the mean curvature and the second fundamental form in a principal curvature direction. It may prove useful for studying embedded metric spaces and could be relevant in manifold learning contexts.

Let *M* be a smooth manifold isometrically embedded in another Riemannian manifold. We propose a family of test measures , where $\sigma , \varepsilon$ are small parameters, whose ‘derivative’ in the 1-Wasserstein distance with respect to variation of the point *x* describes some kind of curvature.

This consideration leads to a novel concept of coarse curvature in the setting of Riemannian submanifolds. Within the applicable range of the parameters, we have an approximation of the mean curvature and the second fundamental form, providing a valuable tool for evaluating these extrinsic curvatures. In more practical applications, we can take test measures built from statistical data and simulations; for instance through the empirical measures of point cloud samples. There is scope for extending to metric embeddings of metric spaces.

In contrast to the intrinsic Riemannian curvature, which characterizes the geometry of a manifold independently of its embedding, the second fundamental form of submanifolds is an extrinsic concept. It provides a means for describing the shape of a submanifold in relation to its ambient space, offering views into its bending properties. For instance, a surface embedded in $\mathbb {R}^3$ is locally isometric to a plane if and only if its second fundamental form vanishes.

The extrinsic curvature of *M*, isometrically embedded in *N*, is expressed by the second fundamental form, which we recall to be defined as the bilinear form1.1where *W* is an arbitrary vector field on *M* with $W(x) = w$. Letting *m* denote the dimension of *M*, the mean curvature is defined as the vector field

$$\begin{aligned} H(x) :=\sum _{i=1}^m \nabla ^N_{e_i} e_i(x) -\nabla ^M_{e_i} e_i(x). \end{aligned}$$

Here $(e_i)_{i=1}^m$ is an arbitrary local orthonormal frame on a neighbourhood of *x* in *M*. Note that we omit the factor of 1/*m* that usually appears in this definition in the literature in order to simplify the statement of our results. It is a standard fact that both  and *H*(*x*) are vectors which are perpendicular to the submanifold *M*. We refer to e.g. [19, Chapter 5] for a detailed treatment of these objects. For instance, one of the examples we consider below is that of a planar curve $\gamma$ with radius of osculating circle $R(\alpha )$. A simple computation shows that in this casewhere $\Vert \hspace{1.111pt}{\cdot }\hspace{1.111pt}\Vert$ is the Euclidean magnitude.

There exists a considerable body of literature on description of submanifold properties by tubular volume, of which we name a few representatives. The early work of Weyl [32] proved the classical tube formula for submanifolds embedded in Euclidean spaces, which is an expansion with respect to the width of the tubular volume and its coefficients are geometric invariants of the submanifold. Federer [10] introduced the notion of boundary measures, which lead to generalization of the tube formula to compact subsets of Euclidean spaces. More recent works of Chazal et al. [5, 6] studied geometric inference via point cloud approximations to boundary measures using Monte Carlo methods. For a comprehensive treatment on properties of tubular neighbourhoods, we refer to the monograph [16]. The approach in our present work differs from the above in that it gives a local and directional information about the second fundamental form, and also the mean curvature.

Notions of synthetic Ricci curvature were motivated by the study of geometry of metric measure spaces and were pioneered by the seminal works [4, 23, 30], see also the survey [22]. In a metric measure space, a global lower bound on the synthetic Ricci curvature leads to properties of the metric measure space which are analogous to the Riemannian setting, such as the Poincaré and log-Sobolev inequalities, the concentration of measure phenomenon, and closure under measured Gromov–Hausdorff convergence [1, 8, 28]. We note also the related direction of the works [4, 31].

To our understanding, there has not been a notion of a synthetic extrinsic curvature. Our notion of coarse extrinsic curvature is inspired by coarse Ricci curvature of Ollivier [25], which is defined in the Riemannian setting through the expansion of the 1-Wasserstein distance of two uniform measures supported on geodesic balls of a small radius, the radius being the variable of expansion [25, Example 7], see also the survey [26]. This is different from the above mentioned synthetic Ricci curvature lower bounds in that it puts a precise number on the value of curvature at a point. Moreover, it can be applied to general metric spaces by choosing a family of measures indexed by points in the space for the evaluation of the 1-Wasserstein distance. Coarse Ricci curvature can be computed explicitly for a number of examples on graphs, where the measures are provided by a Markov chain. We adopt and modify Ollivier’s approach to the submanifold setting by choosing suitable measures for the expansion of the 1-Wasserstein distance, showing that this yields a geometrically meaningful information.

As an immediate application of our result, we venture into the setting of [2, 18] to explore retrieval of curvature information from point clouds generated by a Poisson point process. In the first of the mentioned works, Hoorn et al. proved that Ollivier’s coarse Ricci curvature of random geometric graphs sampled from a Poisson point process with increasing intensity on a Riemannian manifold converges in expectation at every point to the classical Ricci curvature of the manifold. This was extended in the second mentioned work to weighted Riemannian manifolds. In the present work, we show that coarse extrinsic curvature can recover the mean curvature in expectation at a point. In this case, it is not necessary to impose a graph structure to connect points of the sample.

In the context of deep learning, it is noteworthy that computational algorithms for effectively computing optimal transport maps have been proposed, as discussed in [17, 29]. Additionally, relevant work in the fields of manifold learning and inverse problems is worth mentioning. One particularly interesting inverse problem is whether an embedded manifold can be learned from a set of samples $x_j+\xi _j$ where $x_j$ belongs to a submanifold $M\subset \mathbb {R}^{m+p}$ and $\xi _j$ are independent Gaussian random variables on $\mathbb {R}^{m+p}$. The reconstruction of embedded manifolds has been studied in [9, 11, 12, 27]. An algorithm for constructing an embedded submanifold is provided in [13]. Although manifold learning is still in its early stages, manifold approximation and reconstruction have a longer history, we point out some more recent publications on this topic [3, 7, 7, 14, 15].

### Main results

In our setting, *M* is an *m*-dimensional compact Riemannian manifold embedded isometrically in a Euclidean space $\mathbb {R}^{m+k}$ and $M_\sigma$ is the local $\sigma$-tubular neighbourhood of *M* in $\mathbb {R}^{m+k}$, defined for $\sigma$ sufficiently small as

$$\begin{aligned} M_\sigma = \bigl \{x + v\,{:}\, x \in M, v \in T_xM^\perp , \Vert v\Vert \leqslant \sigma \bigr \}. \end{aligned}$$

For any compact subset $U \subset M$, the projection mapping from its tubular neighbourhood

$$\begin{aligned} \pi :U_\sigma \rightarrow U, \quad \pi (z) :=\text {argmin}_{x \in U} \Vert z-x\Vert \end{aligned}$$

is well-defined for all $\sigma >0$ sufficiently small, with the same notation for $U_\sigma$ as above.

Denote by $\exp _{M,x}:T_xM \rightarrow M$ the exponential mapping in *M* with base point *x*. Fix a point $x_0 \in M$, a unit tangent vector $v \in T_{x_0}M$ and denote $y :=\exp _{M,x_0}(\delta v)$ for $\delta >0$. Fix a constant $\varepsilon _0 > 0$ smaller than the uniform injectivity radius of some fixed compact neighbourhood of $x_0$ in *M*. Assume $\delta , \varepsilon < \varepsilon _0 / 3$ so that $B_\varepsilon (x_0) \cup B_\varepsilon (y)$ lie within the uniform injectivity radius away from $x_0$ and assume $\sigma$ is small enough so that the projection $\pi$ is well-defined on the $\sigma$-tubular neighbourhood of the $\varepsilon _0$-geodesic ball at $x_0$ in *M*. These requirements on the parameters $\delta , \varepsilon , \sigma$ will henceforth be encapsulated in the assumption that they are “sufficiently small”. This ensures that all locally defined maps are well-defined, in particular the projection map (smallness of $\sigma$) and the Fermi coordinates (smallness of $\delta$ and $\varepsilon$) used later on.

As our test measures, we choose the probability measureswhere $B^M_\varepsilon (x)$ denotes the $\varepsilon$-geodesic ball at *x* in *M*. Note that these measures are supported on compact subsets of $M_\sigma$. We seek to obtain the expansion of $W_1(\mu _{x_0}^{\sigma ,\varepsilon }, \mu _y^{\sigma ,\varepsilon })$ with respect to the parameters $\delta ,\sigma$ and $\varepsilon$.

To relate the Wasserstein distance to the second fundamental form, we first localize to a tubular neighbourhood of a fixed open set on the submanifold. We expand the densities of the test measures in Fermi coordinates, and for the subsequent computations we rely on a crucial observation developed in Sect. 2.2: if *T* is an approximate transport map from $\mu _{x_0}^{\sigma ,\varepsilon }$ to $\mu _y^{\sigma ,\varepsilon }$, in a sense defined later, then $W_1(\mu _{x_0}^{\sigma ,\varepsilon }, \mu _y^{\sigma ,\varepsilon })$ is close to $W_1(\mu _{x_0}^{\sigma ,\varepsilon }, T_*\mu _{x_0}^{\sigma ,\varepsilon })$. The remaining task involves proposing a concrete approximate transport map, which is at the same time close enough to optimal.

When dealing with test measures on an embedded manifold, accounting for the effect of the bending of the submanifold in the ambient space becomes crucial. The proposed transport map is thus formulated in terms of the Fermi frame along $\gamma$, adapted to the submanifold *M* in a way that separates tangent and normal coordinate directions at every point.

We give a rough outline of the proposed transport map, made precise in Sect. 2.4. In terms of Fermi coordinates, if $\alpha =(\alpha _1,\ldots ,\alpha _m)$ represent submanifold tangent directions with $\alpha _1$ being associated with the direction of $\gamma$, and if $\beta =(\beta _1,\ldots ,\beta _k)$ represent the normal directions, an initial proposal informed by the circle example (Sect. 3.1) was

$$\begin{aligned} (\alpha , \beta ) \mapsto (\delta -\alpha _1, \alpha _2, \dots , \alpha _m, \beta _1,\ldots ,\beta _k). \end{aligned}$$

This can be construed as translation by $\delta$ in the direction of the first coordinate, together with reflection in the first coordinate. From studying the planar curve example (Sect. 3.2), it turned out that an additional bending correction needs to be put on top of the $\beta$ components of the transport by adding terms involving the derivative of the mean curvature. Favourably, such a correction contributes to the final estimate of the Wasserstein distance only at the fourth order and higher, and hence does not interfere with the mean curvature term, which will appear at third order of the expansion. The test measures are first expressed in Fermi coordinates in Sect. 2.3. The proposed transport map is then presented in Sect. 2.4, where we prove that it is indeed an approximate transport map of degree 3, i.e.

$$\begin{aligned} \frac{d(T_* \mu _{x_0}^{\sigma ,\varepsilon })}{d\mu _y^{\sigma ,\varepsilon }}\hspace{0.55542pt}(\phi (\alpha ,\beta )) = 1 + O(\delta ^3). \end{aligned}$$

This precision is sufficient for obtaining the 1-Wasserstein distance approximation (see Sect. 2.2):

$$\begin{aligned} W_1(\mu _{x_0}^{\sigma ,\varepsilon }, \mu _{y}^{\sigma ,\varepsilon }) = W_1(\mu _{x_0}^{\sigma ,\varepsilon }, T_* \mu _{x_0}^{\sigma ,\varepsilon }) + O(\delta ^4). \end{aligned}$$

From here the strategy is to construct a test function  with Lipschitz norm approximately 1 and satisfying the estimate

$$\begin{aligned} f(Tz) - f(z) = \Vert Tz - z\Vert + O(\delta ^4) = O(\delta ), \end{aligned}$$

which allows us to estimate the distance between the original measure and its transport by means of the relation

$$\begin{aligned} W_1(\mu _{x_0}^{\sigma ,\varepsilon }, T_*\mu _{x_0}^{\sigma ,\varepsilon }) = \int (f(Tz) - f(z)) \,d\mu _{x_0}^{\sigma ,\varepsilon }(z) + O(\delta ^4). \end{aligned}$$

On the whole, we find that the Wasserstein distance between the initial measure $\mu _{x_0}^{\sigma ,\varepsilon }$ and the target measure $\mu _y^{\sigma ,\varepsilon }$ is approximated by $\int _M \Vert Tz-z\Vert \hspace{0.55542pt}d\mu _{x_0}^{\sigma ,\varepsilon }$ up to $O(\delta ^4)$ (see Lemma 2.25), which is explicitly computable as an expansion in $\delta , \sigma$ and $\varepsilon$ with geometric quantities as coefficients.

Using the above tools, in Sect. 3 we thus compute the expansion of $W_1(\mu _{x_0}^{\sigma ,\varepsilon }, \mu _y^{\sigma ,\varepsilon })$, beginning with the case of a planar curve:

#### Proposition 1.1

Let $\gamma$ be a smooth unit speed curve in $\mathbb {R}^2$ such that $\gamma (0) = x_0$ and $\gamma (\delta )=y$. For all $\delta , \varepsilon , \sigma >0$ sufficiently small with $\sigma \vee \varepsilon \leqslant {\delta }/{4}$, it holds that

$$\begin{aligned} \begin{aligned} W_1(\mu _{x_0}^{\sigma ,\varepsilon }, \mu _{y}^{\sigma ,\varepsilon })&= \Vert x_0-y\Vert \biggl ( 1-\frac{\varepsilon ^2}{6R^2} + \frac{\sigma ^2}{3R^2} \biggr ) + O(\delta ^4) \end{aligned} \end{aligned}$$

where *R* is the radius of the osculating circle of the curve at $x_0$.

This expansion can be rearranged as

$$\begin{aligned} \begin{aligned} 1- \frac{W_1(\mu _{x_0}^{\sigma ,\varepsilon }, \mu _{y}^{\sigma ,\varepsilon })}{ \Vert x_0-y\Vert }&=\frac{\varepsilon ^2}{6R^2} - \frac{\sigma ^2}{3R^2} + O(\delta ^3). \end{aligned} \end{aligned}$$

We refer to the quantity on the left as the coarse extrinsic curvature of $\gamma$ between $x_0$ and *y* at scales $\sigma ,\varepsilon$. A version of this result for spatial curves is presented in Theorem 3.10. In Theorem 3.16, we then proceed to study the case of coarse extrinsic curvature along a geodesic on a surface embedded in $\mathbb {R}^3$.

This work culminates with the most general form:

#### [Style2 Style3 Style3]Theorem 4.1

Let *M* be an isometrically embedded submanifold of $\mathbb {R}^{m+k}$, and $\gamma$ a unit speed geodesic in *M* such that $\gamma (0) = x_0$ and $\gamma (\delta )=y$. Let $(e_j)_{j=1}^m$ be an orthonormal basis of $T_{x_0}M$ with $e_1 = \dot{\gamma }(0)$ and assume that  for all $j=2,\ldots ,m$. Then for every $\sigma ,\varepsilon , \delta >0$ sufficiently small with $\sigma \vee \varepsilon \leqslant {\delta }/{4}$ it holds that

The assumption on the second fundamental form is necessary for optimality of our proposed transport map up to sufficient order and can always be satisfied for submanifolds of codimension 1, in particular surfaces embedded in $\mathbb {R}^3$, by choosing the basis of principal curvature directions. Further commentary is provided in Remark 4.2.

To interpret such expansions in terms of mean curvature, we can remove the directionality of the above result caused by transport in the direction of $\gamma$. Denoting the square norm of the mean curvature vector as

$$\begin{aligned} \Vert H(x_0)\Vert ^2 = \sum _{i=1}^k\, \langle H(x_0), \textbf{n}_i(x_0)\rangle ^2 \end{aligned}$$

for an arbitrary orthonormal basis $(\textbf{n}_i(x_0))_{i=1}^k$ of the normal space $T_{x_0}M^\perp \subset T_{x_0}N$, we deduce the following:

#### Corollary 1.2

Let $(e_j)_{j=1}^m$ be an orthonormal basis of $\,T_{x_0}M$, and for $j=1, \dots , m$, let $y_j = \exp _{M, x_0}(\delta e_j)$. Assume that  for $i\ne j$. Then for all $\sigma ,\varepsilon ,\delta >0$ sufficiently small with $\sigma \vee \varepsilon \leqslant {\delta }/{4}$ it holds that

$$\begin{aligned} \begin{aligned} \sum _{j=1}^m \biggl ( 1- \frac{W_1(\mu _{x_0}^{\sigma ,\varepsilon }, \mu _{y_j}^{\sigma ,\varepsilon })}{\Vert x_0-y_j\Vert }\biggr )&= \biggl ( \frac{\varepsilon ^2}{2(m+2)} - \frac{\sigma ^2}{k+2} \biggr ) \Vert H(x_0)\Vert ^2 + O(\delta ^3). \end{aligned} \end{aligned}$$

Observe that the left side of the equation is independent of the choice of orthonormal basis $(e_j)_{j=1}^m$ because the norm on the right side is basis-invariant. Moreover, the assumption on the second fundamental form always holds for submanifolds of codimension 1 (see Remark 4.2).

In Proposition 5.4, we deduce that the coarse extrinsic curvature of suitable test measures on Poisson point clouds sampled from the tubular neighbourhood retrieves the same extrinsic geometric information consistent with Theorem 4.1.

One key ingredient in the proofs of the above theorems is the geometric approximate transport map introduced in Definition 2.18, defined by means of Fermi coordinates (as per Definition 2.14) adapted to the submanifold. Test measures in these coordinates encode information about the second fundamental form of the submanifold. The proposed map is verified to be an approximate transport map between the test measures with sufficient order of accuracy, as specified and motivated in Sect. 2.2. The optimality up to fourth order is proved by choosing a concrete test function for the Wasserstein lower bound by the Kantorovich–Rubinstein duality.

In the resulting expansion of the Wasserstein distance, the second fundamental form at the fixed point $x_0$ appears at third order, and its derivatives appear at fourth and higher orders. As a consequence, information about the second fundamental form at a point can be retrieved in a suitably scaled limit of coarse curvature. Please see the discussion below for an example.

#### Discussion

We illustrate this work using the following prototypical example. Let $\gamma :(-\delta _0,\delta _0) \rightarrow \mathbb {R}^2$ be a smooth, unit speed planar curve, and $\textbf{n}:(-\delta _0,\delta _0) \rightarrow \mathbb {R}^2$ a unit normal vector field along $\gamma$, unique up to sign. Denote by $R(\alpha ):=\frac{1}{\Vert \ddot{\gamma }(\alpha )\Vert }$ the radius of the osculating circle at the point $\gamma (\alpha )$. To detect the extrinsic curvature at $x_0:=\gamma (0)$, captured here by *R*(0), we define test probability measures centered at nearby points $y:=\gamma (\delta )$ indexed by $\delta >0$.

Denote $\mu$ the Lebesgue measure on $\mathbb {R}^2$, $M :=\gamma ((-\delta _0,\delta _0))$ as the image of the curve, and $M_{\sigma _0}$ as a small enough tubular neighbourhood of *M* so that the orthogonal projection $\pi :M_{\sigma _0} \rightarrow M$ is well-defined. Denote

$$\begin{aligned} B_{\sigma , \varepsilon }(y) :=\bigl \{z \in \mathbb {R}^2\,{:}\, \Vert z-\pi (z)\Vert< \sigma , d_\gamma (y, \pi (z)) < \varepsilon \bigr \} \end{aligned}$$

where $d_\gamma$ is the distance along $\gamma$. Define for $\sigma , \varepsilon >0$ with $\sigma \vee \varepsilon \leqslant {\delta }/{4}$, the Borel measure on $\mathbb {R}^2$,

$$\begin{aligned} \mu _{y}^{\sigma , \varepsilon }(A) :=\frac{\mu (A \cap B_{\sigma , \varepsilon }(y))}{\mu (B_{\sigma , \varepsilon }(y))}\hspace{0.55542pt}. \end{aligned}$$

We compare these in 1-Wasserstein distance to the initial measure, i.e. when $\delta =0$ and is denoted $\mu _{x_0}^{\sigma , \varepsilon }$. The Wasserstein distance has the form:

$$\begin{aligned} W_1(\mu _{x_0}^{\sigma , \varepsilon }, \mu _y^{\sigma , \varepsilon }) = \Vert x_0-y\Vert \biggl (1- \frac{\varepsilon ^2}{6R(0)^2} +\frac{\sigma ^2}{3R(0)^2}\biggr ) + O(\delta ^4). \end{aligned}$$

Rearranging this expansion yields

1.2 $$\begin{aligned} 1-\frac{W_1(\mu _{x_0}^{\sigma , \varepsilon }, \mu _y^{\sigma , \varepsilon })}{\Vert x_0-y\Vert } = \frac{1}{R(0)^2}\, \biggl (\frac{\varepsilon ^2}{6}-\frac{\sigma ^2}{3}\biggr ) + O(\delta ^3). \end{aligned}$$

From this point, depending on the application, we may consider three different regimes for the parameters $\varepsilon$ and $\sigma$ as *y* converges to $x_0$. We recall the asymptotic notation $\sigma = \Theta (\delta )$ means there exist $c,C,\delta _0 >0$ such that for all $\delta < \delta _0$,

$$\begin{aligned} c \delta< \sigma (\delta ) < C \delta , \end{aligned}$$

and $$\sigma = o(\delta )$$ means $$\lim _{\,\delta \rightarrow 0} \frac{\sigma (\delta )}{\delta } = 0$$.

(i) $\lim _{\,\delta \rightarrow 0} \frac{\varepsilon (\delta )}{\sigma (\delta )} = C \ne \sqrt{2}$ for some known constant $C >0$, i.e. the decay of both $\sigma$ and $\varepsilon$ is controlled. In this case,
  $$\begin{aligned} \frac{1}{R(0)^2} = \lim _{\delta \rightarrow 0} -\frac{6}{(C^2-2)\hspace{0.55542pt}\sigma ^2}\, \biggl ( 1- \frac{W_1(\mu _{x_0}^{\sigma , \varepsilon }, \mu _y^{\sigma , \varepsilon })}{\Vert x_0-y\Vert } \biggr ), \end{aligned}$$
(ii) $\sigma = \Theta (\delta )$ and $\varepsilon = o(\delta )$, i.e. the decay of $\sigma$ is controlled, while the parameter of support size $\varepsilon$ vanishes fast. In this case,
  $$\begin{aligned} \frac{1}{R(0)^2} = \lim _{\begin{array}{c} \varepsilon = o(\sigma ), \sigma = \Theta (\delta ) \\ \delta \rightarrow 0 \end{array}} -\frac{3}{\sigma ^2}\, \biggl ( 1- \frac{W_1(\mu _{x_0}^{\sigma , \varepsilon }, \mu _y^{\sigma , \varepsilon })}{\Vert x_0-y\Vert } \biggr ), \end{aligned}$$
(iii) $\varepsilon = \Theta (\delta )$ and $\sigma = o(\delta )$, i.e. the decay of $\varepsilon$ is controlled, while the size of the tubular neighbourhood $\sigma$ vanishes fast. In this case,
  $$\begin{aligned} \frac{1}{R(0)^2}= \lim _{\begin{array}{c} \sigma = o(\varepsilon ), \varepsilon = \Theta (\delta )\\ \delta \rightarrow 0 \end{array}} \frac{6}{\varepsilon ^2}\, \biggl ( 1- \frac{W_1(\mu _{x_0}^{\sigma , \varepsilon }, \mu _y^{\sigma , \varepsilon })}{\Vert x_0-y\Vert } \biggr ). \end{aligned}$$

The requirements $\sigma = \Theta (\delta )$ and $\varepsilon = \Theta (\delta )$ in the respective cases are in place to ensure the remainder term $O(\delta ^3)$ in (1.2) does not explode upon division by $\sigma ^2$ (resp. $\varepsilon ^2$) in the limit as $\delta \rightarrow 0$.

In light of the above discussion, we may define the coarse extrinsic curvature between $x_0$ and *y* at scales $\varepsilon , \sigma >0$ as:

1.3 $$\begin{aligned} \kappa _{\sigma , \varepsilon }(x_0,y) :=1-\frac{W_1(\mu _{x_0}^{\sigma , \varepsilon }, \mu _y^{\sigma , \varepsilon })}{\Vert x_0-y\Vert }\hspace{0.55542pt}. \end{aligned}$$

This quantity can be estimated from point cloud data and used for geometric inference.

In summary, this work focuses on Riemannian submanifolds embedded isometrically in Euclidean spaces with the aim of producing a reasonable measurement for the bending energy. This bending energy can also be estimated from point clouds obtained from sampling. One of the novel ingredients is the construction of a test function for using the Kantorovich–Rubinstein duality to obtain a lower bound for the Wasserstein distance in this setting.

The outline of this work is as follows. In Sect. 2, we establish geometric preliminaries pertaining to the volumes of tubular neighbourhoods and present approximate transport maps as a novel tool for approximating the 1-Wasserstein distance. In Sect. 3, we give description of coarse extrinsic curvature for a planar curve, space curve and a 2-surface embedded in $\mathbb {R}^3$. The coarse extrinsic curvature of a general submanifold of arbitrary codimension is studied in Sect. 4. We present several immediate corollaries to our results with practical applications in Sect. 5. Although the cases of curves and surfaces in Sect. 3 are just instances of the general result in Sect. 4, they provide value in understanding this general case. Sections 3 and 4 can be read separately after reading Sect. 2, which contains all preliminaries.

## Preliminaries

We prove a formula for volume growth of tubular neighbourhoods of submanifolds, leading to a disintegration of the ambient volume measure adapted to the submanifold. This formula is subsequently utilized to derive explicit formulas for such disintegration in Fermi coordinates, considering cases such as a planar curve, space curve, and a surface in Sect. 3, and general Riemannian submanifolds in Sect. 4.

Following the geometric preliminaries, we introduce the notion of an approximate transport map, enabling the computation of Wasserstein distances up to a sufficiently high degree of error. Subsequently, we define the test measures to be transported and their representation in Fermi coordinates. Finally, we propose a transport map to evaluate the Wasserstein distance of these test measures.

### Ambient volume disintegration

We begin with a simple lemma on evolution of probability densities. We denote  as the space of probability measures on a measurable space . The notation $\mu \ll \nu$ denotes the fact that the measure $\mu$ is absolutely continuous with respect to the measure $\nu$.

#### Lemma 2.1

Consider  such that $\mu _t \ll \mu _s$ for all $s\leqslant t$. Let , $t \geqslant 0,$ be a family of functions with $t \mapsto h_t(x)$ locally integrable, and such that $\frac{d}{ds}\big |_{s=0}\frac{d\mu _{t+s}}{d\mu _t}(x) = h_t(x)$ for every $t \geqslant 0$. Then

$$\begin{aligned} \frac{d\mu _t}{d\mu _0}\hspace{0.55542pt}(x) = e^{\int _0^t h_s(x)\hspace{0.55542pt}ds}. \end{aligned}$$

#### Proof

The change of density at any $t \geqslant 0$ satisfies

$$\begin{aligned} \frac{d}{ds}\bigg |_{s=0}\frac{d\mu _{t+s}}{d\mu _t}\hspace{0.55542pt}(x) = \frac{d}{ds}\bigg |_{s=0}\frac{\frac{d\mu _{t+s}}{d\mu _0}(x)}{\frac{d\mu _t}{d\mu _0}(x)}=h_t(x) \end{aligned}$$

implying

$$\begin{aligned} \frac{d}{ds}\bigg |_{s=0}\frac{d\mu _{t+s}}{d\mu _0}\hspace{0.55542pt}(x) = h_t(x) \,\frac{d\mu _t}{d\mu _0}\hspace{0.55542pt}(x) \end{aligned}$$

which has the unique solution $\frac{d\mu _t}{d\mu _0}(x) = e^{\int _0^t h_s(x)\hspace{0.55542pt}ds}$ by standard ODE theory. $\square$

#### Notation 2.2

Throughout this article, *M* is a compact Riemannian manifold of dimension *m*, isometrically immersed in a Riemannian manifold *N* of dimension *n*. Set $k:=n-m$. Let $\sigma _0>0$ be a fixed number smaller than half the reach of *M* in *N*. The reach is defined as the maximal number *r* such that each point within a distance *r* from *M* has a unique orthogonal projection to *M*, . The projection map is well-defined within the ‘reach’.

Let $U \subset M$ be a sufficiently small open neighbourhood such that there exists an orthonormal frame of unit normal vector fields $(\textbf{n}_1, \ldots , \textbf{n}_k)$ on *U* and a one-parameter family of vector fields $\{(e_i(s))_{i=1}^{m}\,{:}\, s\in (-\sigma _0, \sigma _0)\}$ such that $(e_i(s))_{i=1}^m$ is an orthonormal frame on $\psi _s(U)$ for every $s \in (-\sigma _0,\sigma _0)$, and $s \mapsto e_i(s)$ is smooth for every $i=1,\ldots ,m$. The latter can be constructed by taking the pushforward of an arbitrary initial orthonormal frame by $\psi _s$, denoted by $(D_{e_i(0)} \psi _s)_{i=1}^m$, and applying the Gram–Schmidt orthonormalization procedure.

#### Definition 2.3

Let $\textbf{n}\in \Gamma (TU^\perp )$ be a unit normal vector field, and define the normal flow $\psi :M \hspace{1.111pt}{\times }\hspace{1.111pt}(-2\sigma _0, 2\sigma _0) \rightarrow N$ by

$$\begin{aligned} \psi _t(x) :=\exp _{N,x} (t\textbf{n}(x)) \end{aligned}$$

where $\exp _{N,x}:T_xN \rightarrow N$ denotes the exponential mapping on *N*. Denote by  the parallel transport with respect to the Levi-Civita connection $\nabla ^N$ along $t \mapsto \psi _t(x)$ for a fixed $x\in M$, and note that .

For every $t \in (-2\sigma _0, 2\sigma _0)$, $\psi _t$ is a diffeomorphism onto its image, and $t\mapsto \psi _t(x)$ is smooth with non-vanishing derivative for every $x\in M$. Equip every $\psi _t(M)$ with the Riemannian metric inherited from the ambient space. The mean curvature of the leaf $\psi _s(U)$ is then given by

$$\begin{aligned} H(\psi _s(x)) = \sum _{i=1}^m \nabla ^N_{e_i} e_i(\psi _s(x)) - \nabla ^M_{e_i} e_i(\psi _s(x)). \end{aligned}$$

In particular, for any unit normal vector field $\textbf{n}$ on *U*,

The following lemma shows  stays normal to the leaves $\psi _t(U)$ as *t* changes.

#### Lemma 2.4

The vector field  is normal to $\psi _t(U)$ for every $t \in (-\sigma _0, \sigma _0)$, i.e.  for any local tangent frame $(e_i)_{i=1}^m$ on *M*.

#### Proof

For every $t \in (-\sigma _0, \sigma _0)$, $\psi _t$ being a diffeomorphism implies that if $(e_i)_{i=1}^m$ is a frame on *U*, then $(D_{e_i} \psi _t)_{i=1}^m$ is a frame on $\psi _t(U)$, not necessarily orthonormal. Thenwhere on the second line we used that . The initial condition $\psi _0 = {\text {id}}$ gives $\langle D_{e_i} \psi _0, \textbf{n} \rangle = \langle e_i, \textbf{n} \rangle = 0$, so we may conclude that  is normal to all tangent directions on $\psi _t(U)$ for all *t*. $\square$

The action of push-forwards of volume forms on any orthonormal basis of tangent vectors is characterized by the determinant of the mapping which we make precise below. Let $M_1, M_2$ be Riemannian manifolds of the same dimension *m*, $\psi :M_1 \rightarrow M_2$ a diffeomorphism, $(e_i)_{i=1}^m$ an orthonormal frame on an open set $U_1 \subset M_1$ and $(\tilde{e}_i)_{i=1}^m$ an orthonormal frame on an open set $U_2 \subset M_2$, and $(e^i)_{i=1}^m, (\tilde{e}^i)_{i=1}^m$ the corresponding coframes characterized by $e^i(e_j) = \delta ^i_j, \tilde{e}^i(\tilde{e}_j) = \delta ^i_j$. Below, by the determinant of $D\psi ^{-1} (x) :T_x M_2\rightarrow T_{\Psi ^{-1}(x)}M_1$ we mean that of the matrix representing the map in these bases:

$$\begin{aligned} \det D\psi ^{-1}=\sum _{\sigma \in S_m} \prod _{i=1}^m {\mathop {\textrm{sign}}}\hspace{0.55542pt}(\sigma ) \hspace{1.111pt}e^i\bigl (\psi ^{-1}_* \tilde{e}_{\sigma (i)}\bigr ). \end{aligned}$$

By the rules of differential forms acting on tangent vectors, for all $x \in U_2$,

$$\begin{aligned} \begin{aligned} \psi _* (e^1 \hspace{0.55542pt}{\wedge }\hspace{1.111pt}\cdots \hspace{1.111pt}{\wedge }\hspace{1.111pt}e^m)(x)&(\tilde{e}_1(x),\ldots , \tilde{e}_m(x))\\&= (e^1 (x)\hspace{1.111pt}{\wedge }\hspace{1.111pt}\cdots \hspace{1.111pt}{\wedge }\hspace{1.111pt}e^m(x))(\psi ^{-1}_* \tilde{e}_1(x),\ldots , \psi ^{-1}_* \tilde{e}_m(x)) \\&= \sum _{\sigma \in S_m} \prod _{i=1}^m {\mathop {\textrm{sign}}}\hspace{0.55542pt}(\sigma )\hspace{1.111pt}e^i(x)\bigl (\psi ^{-1}_* \tilde{e}_{\sigma (i)(x)}\bigr ) \\&= \det D\psi ^{-1}. \end{aligned} \end{aligned}$$

Since linear maps are determined by their values on basis vectors, we may deduce

2.1 $$\begin{aligned} \psi _* (e^1 \hspace{0.55542pt}{\wedge }\hspace{1.111pt}\cdots \hspace{1.111pt}{\wedge }\hspace{1.111pt}e^m)(x) = \det D\psi ^{-1}(x) \hspace{1.111pt}\tilde{e}^1 \hspace{0.55542pt}{\wedge }\hspace{1.111pt}\cdots \hspace{1.111pt}{\wedge }\hspace{1.111pt}\tilde{e}^m (x). \end{aligned}$$

With the above notation we return to the exponential map $\psi _t(x) :=\exp _{N,x} (t\textbf{n}(x))$.

#### Proposition 2.5

(Change of volume) For every $t \in (-\sigma _0, \sigma _0)$,2.2and hence the volume of the image of any Borel measurable $A \subset U$ can be expressed as2.3where $\textrm{vol}_{\psi _t(M)}$ is the Riemannian volume on $\psi _t(M)$.

#### Proof

First, we extend the map $\psi :(-\sigma _0,\sigma _0) \hspace{1.111pt}{\times }\hspace{1.111pt}U \rightarrow N$ to $\tilde{\psi }:(-\sigma _0,\sigma _0) \hspace{1.111pt}{\times }\hspace{1.111pt}U_{\sigma _0} \rightarrow N$ by the flow condition

$$\begin{aligned} \tilde{\psi _s}(\psi _t(x)) :=\psi _{t+s}(x) \end{aligned}$$

for all *s* and *t* in $(-\sigma _0,\sigma _0)$. This determines $\tilde{\psi }$ uniquely because $\{\psi _t(U)\}_{t \in (-\sigma _0,\sigma _0)}$ is a foliation of the tubular neighbourhood $U_{\sigma _0}$. Then on every leaf $\psi _t(U)$ of the foliation, we have $\tilde{\psi }_0 = {\text {id}}$. If $(e^i)_{i=1}^m$ and $(\tilde{e}^i)_{i=1}^m$ are orthonormal coframes on $\psi _{t+s}(U)$ and $\psi _t(U)$ respectively, the change of variable formula for volume forms (2.1) states that

$$\begin{aligned} (\tilde{\psi }_{-s})_* \hspace{1.111pt}e^1 \hspace{0.55542pt}{\wedge }\hspace{1.111pt}\cdots \hspace{1.111pt}{\wedge }\hspace{1.111pt}e^n = (\det D \tilde{\psi }_s) \hspace{1.111pt}\tilde{e}^1 \hspace{0.55542pt}{\wedge }\hspace{1.111pt}\cdots \hspace{1.111pt}{\wedge }\hspace{1.111pt}\tilde{e}^n. \end{aligned}$$

Then for every $A \subset U$ Borel measurable and $s \in (-\sigma _0,\sigma _0)$,

$$\begin{aligned} \begin{aligned} \textrm{vol}_{\psi _{t+s}(M)}(\psi _{t+s}(A))&= \textrm{vol}_{\psi _{t+s}(M)}(\tilde{\psi }_s(\psi _{t}(A))) \\&= \bigl ((\tilde{\psi }_{-s})_* \textrm{vol}_{\psi _{t+s}(M)}\bigr ) (\psi _t(A)) \\&= \int _{\psi _t(A)} \det D \tilde{\psi }_s(x) \,d \textrm{vol}_{\psi _t(M)}(x) \end{aligned} \end{aligned}$$

using respectively the flow property, definition of the push-forward of a measure, and the change of variable formula with $\textrm{vol}_{\psi _{t+s}(M)}= e^1 \hspace{0.55542pt}{\wedge }\hspace{1.111pt}\cdots \hspace{1.111pt}{\wedge }\hspace{1.111pt}e^m$ and $\textrm{vol}_{\psi _{t}(M)} = \tilde{e}^1 \hspace{0.55542pt}{\wedge }\hspace{1.111pt}\cdots \hspace{1.111pt}{\wedge }\hspace{1.111pt}\tilde{e}^m$.

Denoting by $\frac{D}{\partial s}$ the covariant derivative along $s \mapsto \tilde{\psi }_s$, the Jacobi formula for the derivative of determinants givesFrom the second to third line, the other term coming from the product rule applied on the bracket does not contribute to the trace, because for $i=j$,

$$\begin{aligned} \biggl \langle D_{e_i(t)} \tilde{\psi }_0, \frac{D}{dt}\hspace{0.55542pt}e_i(t) \biggr \rangle = \biggl \langle e_i(t), \frac{D}{dt}\hspace{0.55542pt}e_i(t) \biggr \rangle = \frac{1}{2} \,\frac{\partial }{\partial t}\hspace{0.55542pt}\langle e_i(t), e_i(t)\rangle = 0 \end{aligned}$$

using that $\tilde{\psi }_0(x)=x$ so $D \tilde{\psi }_0 = \text {id}$. From the fourth to fifth line, we used normality of the flow $\langle \frac{\partial }{\partial s}|_{s=0} \tilde{\psi }_s(x), e_i(t)(x)\rangle = 0$, and on the last line applied $\frac{\partial }{\partial s}\tilde{\psi }_s(\psi _t(x)) = \frac{\partial }{\partial s}\psi _{t+s}(x)$ from definition of the extension $\tilde{\psi }$, before pulling the integral from $\psi _t(A)$ back to *A*.

Hence the evolved volume measure pulled back to *U* satisfies the dynamics2.4which together with the initial condition $d(\psi _0)^{-1}_*\textrm{vol}_{\psi _0(M)} = d\textrm{vol}_M$ impliesby Lemma 2.1, setting $h_t$ to be the right-hand side of (2.4). Equation (2.3) is then simply the change of variable formula for the map $\psi _t$. $\square$

#### Remark 2.6

The formula of Proposition 2.5 can be extended from the neighbourhood *U* to all of *M* by a partition of unity argument, nonetheless the local formulation is sufficient for our purpose.

We proceed to derive a disintegration of the ambient volume measure adapted to a submanifold of arbitrary codimension, at the cost of specialising to the case $N = \mathbb {R}^n$. Note that the covariant derivative $\nabla ^{\mathbb {R}^n}$ then becomes the plain derivative denoted by *D*.

#### Notation 2.7

Let $(\textbf{n}_j)_{j=1}^k$ be a local orthonormal frame for $TM^\perp$ on *U* and denote  the centered Euclidean ball of radius $\sigma _0$. Define the map2.5which gives the *k*-dimensional foliation $\{\psi (U, \beta ) \,{:}\, \beta \in B_{\sigma _0}^k\}$ of $\pi ^{-1}(U)$ with leaves of dimension *m*. Extend $(\textbf{n}_j)_{j=1}^k$ and $(e_i)_{i=1}^m$ smoothly to $\pi ^{-1}(U)$ so that the restrictions to the submanifold $\psi (U,\beta )$ are an orthonormal frame in the tangent space and the normal space, respectively, for every $\beta \in \widetilde{B}_{\sigma _0}^k$.

Denote $\textbf{n}(x,\beta ) = \sum _{j=1}^k \frac{\beta _j \textbf{n}_j(x)}{\Vert \beta \Vert _2}$ which was shown in Lemma 2.4 to be normal to each leaf $\psi (U,\beta )$. Then the mean curvature of $\psi (U,\beta )$ in the direction $\textbf{n}(x,\beta )$ is

$$\begin{aligned} \langle H(\psi (x, \beta )), \textbf{n}(x,\beta )\rangle = \biggl \langle \textbf{n}(x,\beta ), \sum _{i=1}^m \nabla ^{\mathbb {R}^{m+k}}_{e_i} e_i (\psi (x, \beta )) \biggr \rangle . \end{aligned}$$

Denote also the components of mean curvature in each of the directions of the normal frame,

$$\begin{aligned} H^j(\psi (x,\beta )) :=\langle \textbf{n}_j(x), H(\psi (x,\beta )) \rangle = \biggl \langle \textbf{n}_j(x), \sum _{i=1}^m \nabla ^{\mathbb {R}^{m+k}}_{e_i} e_i(\psi (x,\beta )) \biggr \rangle \end{aligned}$$

so that

$$\begin{aligned} \Vert \beta \Vert \langle H(\psi (x, \beta )), \textbf{n}(x,\beta )\rangle = \sum _{j=1}^k \beta _j H^j(\psi (x,\beta )). \end{aligned}$$

#### Remark 2.8

The collection of submanifolds $\{\psi (U,\beta )\,{:}\, \beta \in \widetilde{B}_{\sigma _0}^k\}$ is indeed a foliation of $\pi ^{-1}(U) \subset \mathbb {R}^{m+k}$ (see e.g. the definition of foliation in [21]). The leaves are disjoint submanifolds of dimension *m*. Defining

$$\begin{aligned} F:(p_1,\ldots ,p_m, \beta _1,\ldots , \beta _k) \mapsto \xi (p_1,\ldots ,p_m) + \sum _{j=1}^k \beta _j \textbf{n}_j(\xi (p_1,\ldots ,p_m)) \end{aligned}$$

where $\xi :O \subset \mathbb {R}^m \rightarrow U$ is an arbitrary chart on *U*, we have by definition that

$$\begin{aligned} \psi (U,\beta ) = F(\{\beta \}), \end{aligned}$$

so each leaf is a level set of *F* and thus *F* is a flat chart for the foliation.

#### Proposition 2.9

(Disintegration) The ambient volume measure on $\pi ^{-1}(U) \subset \mathbb {R}^n$ disintegrates with respect to the submanifold and the normal frame $(\textbf{n}_j)_{j=1}^k$ as2.6for any Borel measurable set  in the tubular neighbourhood.

#### Proof

We apply the change of coordinates by the map defined by (2.5), which at every $(x,\beta )$ has block-triangular derivative with respect to the orthonormal bases

$$\begin{aligned} \bigl (e_1(x), \ldots , e_m(x), \partial _{\beta _1}(x), \ldots , \partial _{\beta _k}(x)\bigr ) \end{aligned}$$

and

$$\begin{aligned} \bigl (e_1(\psi (x,\beta )), \ldots , e_m(\psi (x,\beta )), \textbf{n}_1(x), \ldots , \textbf{n}_k(x)\bigr ) \end{aligned}$$

in the domain and codomain respectively, since

$$\begin{aligned} \langle \partial _{\beta _i} \psi (x,\beta ), e_j(x,\beta ) \rangle = \langle \textbf{n}_i(x), e_j(\psi (x,\beta )) \rangle = 0. \end{aligned}$$

Hence the determinant can be computed as

$$\begin{aligned} \begin{aligned} \det D\psi&= \det \hspace{0.55542pt}(\langle D_{e_i} \psi , e_j \rangle ) \det \hspace{0.55542pt}( \langle \partial _{\beta _i} \psi , \textbf{n}_j \rangle ) \\&= \det \hspace{0.55542pt}(\langle D_{e_i} \psi , e_j \rangle ) \det \hspace{0.55542pt}(\delta ^i_j) \\&= \det \hspace{0.55542pt}(\langle D_{e_i} \psi , e_j \rangle ) \end{aligned} \end{aligned}$$

for which we have the right-hand side of (2.2).

Denoting $(e^i)_{i=1}^m$, $(\textbf{n}^i)_{i=1}^k$ the coframes characterized by $e^i(e_j) = \delta ^i_j$ and $\textbf{n}^i(\textbf{n}_j) = \delta ^i_j$,on the second line using the change of variable formula and on the third line plugging in the determinant expression (2.2) with $\textbf{n}(x, \beta ) = \sum _{j=1}^k \frac{\beta _j \textbf{n}_j(x)}{\Vert \beta \Vert }$ and $t = \Vert \beta \Vert _2$. The final expression is obtained by the substitution $s' = \frac{s}{\Vert \beta \Vert }$ so that$\square$

#### Corollary 2.10

(Codimension 1) If *M* has codimension 1 then the ambient volume measure on $\pi ^{-1}(U)$ can be written in terms of the disintegration

2.7 $$\begin{aligned} \textrm{vol}_{\hspace{1.111pt}\mathbb {R}^n}(A) = \int _{U} \int _{-\sigma }^{\sigma } \mathbb {1}_A(\psi (x,\beta ))\hspace{1.111pt}e^{-\int _0^\beta \langle H(\psi (x, \beta '), \textbf{n}(x,\beta ')\rangle d\beta '} d\beta \; \textrm{vol}_M(dx), \end{aligned}$$

for all , where $\textrm{vol}_M(dx)$ is the volume measure of the submanifold *M* and $H(\psi (\hspace{1.111pt}{\cdot }\hspace{1.111pt}, \beta ))$ is the mean curvature on the Riemannian submanifold $\psi (U,\beta )$.

### Approximate transport maps

In the sequel, we work with transport maps which are only optimal up to sufficiently high degree for asymptotically small diameter of support of the test measures. We present a result which justifies the use of such transport maps.

Let  be a Polish space,  the set of probability measures, defineand consider two families of probability measures , .

#### Lemma 2.11

($W_1$ distance approximation) If $\textrm{diam}\hspace{1.111pt}\textrm{supp} \, \mu _2^\delta = O(\delta ^\ell )$ and $\mu _1^{\delta } \ll \mu _2^\delta$ for every $\delta \geqslant 0$ with the density satisfying $\sup _{x \in \textrm{supp}\,\mu _2} \frac{d\mu _1^\delta }{d\mu _2^\delta }(x) = 1+O(\delta ^k)$, then

#### Proof

By the reverse triangle inequality and Kantorovich–Rubinstein duality, for all :where $x_0 \in \text {supp}\, \mu _2^\delta$ is arbitrary. On the last line, we introduced the termbecause $f(x_0)$ is constant and the density integrates to 1, and then used the 1-Lipschitz property of *f* together with the $O(\delta ^\ell )$ bound on the diameter of the support of $\mu _2$. $\square$

Let  be another family of probability measures.

#### Definition 2.12

(Approximate transport map) A measurable map  is said to be an *approximate transport* from $\mu ^\delta$ to $\mu _2^\delta$ with degree *k* if $T^\delta _* \mu ^\delta \ll \mu _2$ and the density satisfies

#### Corollary 2.13

If  is an approximate transport map from $\mu ^\delta$ to $\mu _2^\delta$ with degree *k* and $\textrm{diam}\hspace{1.111pt}\textrm{supp}\, \mu _2^\delta = O(\delta ^\ell )$ then

#### Proof

Set $\mu _1^\delta :=T_*^\delta \mu ^\delta$ and apply the previous lemma. $\square$

### Test measures in Fermi coordinates

Let (*M*, *g*) be a Riemannian submanifold of codimension *k* in $\mathbb {R}^{m+k}$ and $U \subset M$ an open neighbourhood of a point $x_0 \in M$ as in Notation 2.2. The Fermi coordinates are a suitable tool for explicit computations and will be used throughout the rest of this work. The following is a modification of classical Fermi coordinates to the submanifold setting.

#### Definition 2.14

(Fermi coordinates) Let $\gamma :(-\delta _0,\delta _0) \rightarrow M$ be a unit speed geodesic with $\delta _0 >0$ small enough for $\gamma$ to be contained in *U*, $\varepsilon _0$ the uniform injectivity radius in *M* along $\gamma$ and $\sigma _0$ smaller than half the reach of *U* in $\mathbb {R}^{m+k}$. Let $(e_i)_{i=1}^m$ be an orthonormal frame for the fibres of *TM* along $\gamma$ such that $e_1(\alpha _1) = \dot{\gamma }(\alpha _1)$ and $\nabla ^M_{\dot{\gamma }} e_i (\alpha _1) = 0$ for $i=1,\ldots ,m$ and every $\alpha _1 \in (-\delta _0,\delta _0)$. Also let $(\textbf{n}_i)_{i=1}^k$ be a local orthonormal frame for fibres of the normal bundle $TM^\perp$ along $\gamma$.

Denote by $\widetilde{B}_\varepsilon ^{m-1}$ the centered ball of radius $\varepsilon >0$ in $\mathbb {R}^{m-1}$ and by $\widetilde{B}_\sigma ^k$ the centered ball of radius $\sigma > 0$ in $\mathbb {R}^k$. Denote $\alpha =(\alpha _1,\ldots ,\alpha _m), \beta =(\beta _1,\ldots ,\beta _k)$ and define

$$\begin{aligned} \begin{aligned}&\phi :(-\delta _0,\delta _0) \hspace{1.111pt}{\times }\hspace{1.111pt}\widetilde{B}_{\varepsilon _0}^{m-1} \hspace{1.111pt}{\times }\hspace{1.111pt}\widetilde{B}_{\sigma _0}^k \rightarrow \mathbb {R}^{m+k}, \\ \phi (\alpha ,\beta )&:=\exp _{M, \gamma (\alpha _1)} \biggl (\sum _{i=2}^m \alpha _i e_i(\alpha _1)\biggr ) + \sum _{j=1}^k \beta _j \textbf{n}_j(\alpha ), \end{aligned} \end{aligned}$$

which is a diffeomorphism provided that $\delta _0,\varepsilon _0,\sigma _0 > 0$ are sufficiently small. This is referred to as the Fermi chart along $\gamma$ adapted to the submanifold *M*. See Fig. 3 for an illustration on a 2-surface in $\mathbb {R}^3$.

The Riemannian metric is expressed in the Fermi coordinates as

2.8 $$\begin{aligned} g_{ij}(\alpha ) = \langle \partial _{\alpha _i} \phi (\alpha ,0), \partial _{\alpha _j} \phi (\alpha ,0) \rangle . \end{aligned}$$

#### Remark 2.15

The advantage of $\phi$ over a generic $\psi$ as given in Notation 2.7 is that $\phi$ is adapted to the geodesic $\gamma$ in a way that simplifies computations of distances relevant to our optimal transport problem. The chart $\phi$ yields again a foliation $\{\phi (U, \beta ) \,{:}\beta \in \widetilde{B}_{\sigma _0}^k\}$ of $M_{\sigma _0}$.

#### Definition 2.16

(Test measures) Denote the cylinder-like segment in $\mathbb {R}^n$ of height $\sigma$ and radius $\varepsilon$ centered at $x \in M$ as

$$\begin{aligned} B_{\sigma ,\varepsilon }(x) :=\{z \in M_\sigma \,{:}\, d_M(\pi (z), x) < \varepsilon \} \end{aligned}$$

and let $\mu$ be the Lebesgue measure on $\mathbb {R}^n$. For any $x \in M$ define the family of *test probability measures*indexed by $\varepsilon , \sigma >0$.

Denote $\hat{\alpha } = (\alpha _2,\ldots ,\alpha _m)$ so that $\alpha = (\alpha _1, \hat{\alpha })$. The main purpose of the expansion in the following lemma is twofold. First, we use it to design the third order corrections in the approximate transport map of Definition 2.18 so that density matching occurs in Proposition 2.23. Second, the first order term of the expansion interacts with first order term of pointwise distance when integrating to get the Wasserstein upper bound in the proofs of Sects. 3 and 4.

#### Lemma 2.17

(Test measures in Fermi coordinates) For any $y = \gamma (\delta )$, the expansion of the density of test measures in Fermi coordinates is

2.9 $$\begin{aligned} \begin{aligned}&(\phi _*^{-1} \mu _y^{\sigma , \varepsilon })(d\alpha , d\beta )\\&\;\;= \frac{1}{Z} \,\mathbb {1}_{\tilde{B}_{\sigma ,\varepsilon }}(\delta +\alpha _1, \hat{\alpha },\beta ) \biggl ( 1 - \sum _{i=1}^k \beta _i H^i(\phi (\textbf{0})) - \sum _{i=1}^k \sum _{j=1}^m \alpha _j \beta _i \partial _{\alpha _j}(H^i \hspace{0.55542pt}{\circ }\hspace{1.111pt}\phi )(\textbf{0})\\&\qquad \quad - \sum _{i,j=1}^k \beta _i \beta _j \partial _{\beta _j} (H^i \hspace{0.55542pt}{\circ }\hspace{1.111pt}\phi )(\textbf{0}) + \frac{1}{2} \sum _{i,j=1}^k \beta _i \beta _j H^i(\phi (\textbf{0})) H^j(\phi (\textbf{0}))\\&\qquad \quad + \frac{1}{4} \sum _{q,\ell =2}^m \sum _{i=1}^m\alpha _q \alpha _\ell \partial _{\alpha _q}\partial _{\alpha _\ell } g_{ii}(\textbf{0}) + O(\delta ^3) \biggr )\, d\alpha \hspace{1.111pt}d\beta \end{aligned} \end{aligned}$$

where *Z* is the probability normalization constant and $g=(g_{ij})$ is the Riemannian metric of *M* in Fermi coordinates given by (2.8).

#### Proof

First, note the pull-back of the test measure $\mu _y^{\sigma , \varepsilon }(d\alpha , d\beta )$ to Fermi coordinates isThe Riemannian metric in Fermi coordinates expands as2.10Indeed, $\partial _{\alpha _i} g_{j\ell }(\alpha _1,\textbf{0}) = 0$ for all $\alpha _1 \in (-\delta _0,\delta _0)$, and hence also $\partial _{\alpha _1} \partial _{\alpha _i} g_{j\ell }(\alpha _1,\textbf{0}) = 0$. We show this by cases:

- for all $i,j,\ell = 2,\ldots , m$: $\partial _i g_{j\ell }(\alpha _1, \textbf{0}) = 0$ because for every fixed $\alpha _1 \in (-\delta _0,\delta _0)$, $\phi (\alpha _1, \hspace{0.55542pt}{\cdot }\hspace{1.111pt})$ are normal coordinates within the injectivity radius of $\exp _{\gamma (\alpha _1)} (\{\dot{\gamma }(\alpha _1)\}^\perp )\subset M$ at $\gamma (\alpha _1)$ (see e.g. [19, Section 1.4] for a proof),
- for all $i,j=1,\ldots ,m$: $\partial _1 g_{ij}(\alpha _1, \textbf{0}) = 0$ for any $\alpha _1 \in (-\delta _0,\delta _0)$ as the orthonormal frame along $\gamma$ used to define the Fermi chart is parallel translated along $\gamma$,
- for all $i=2,\ldots ,m$ and $j=1,\ldots ,m$ and any $\alpha _1 \in (-\delta _0,\delta _0)$:  using that $\nabla ^{\mathbb {R}^n}_{\partial _{\alpha _1} \phi } \partial _{\alpha _i} \phi (\alpha _1,\textbf{0}) - \nabla ^M_{\partial _{\alpha _1} \phi } \partial _{\alpha _i} \phi (\alpha _1,\textbf{0}) \perp M$ and $\nabla ^M_{\partial _{\alpha _i}\phi } \partial _{\alpha _j} \phi (\alpha _1,\textbf{0}) = 0$. The latter vanishes for $j\ne 1$ again by normality of the chart on $\exp _{\gamma (\alpha _1)} (\{\dot{\gamma }(\alpha _1)\}^\perp )$, and for $j = 1$ because $\partial _{\alpha _i} \phi$ is given by parallel translation along $\gamma$.

The Riemannian volume expanded in the Fermi coordinates then simplifies toMoreover, expand the exponent in the normal part of the disintegration asand apply the approximation up to second order $e^x = 1+x+\frac{x^2}{2} + O(x^3)$ to obtain2.11We conclude the result by taking the product of the two factors (2.10) and (2.11), merging third order terms in $\alpha ,\beta$ into $O(\delta ^3)$ by the assumption $\varepsilon \vee \sigma \leqslant {\delta }/{4}$.

The probability normalization constant can be deduced by integration of (2.11) with respect to $\mu _y^{\sigma ,\varepsilon }$ as

$$\begin{aligned} Z = 1 + \frac{1}{2(k+2)} \,\sigma ^2 \sum _{i=1}^k H^i(\phi (\textbf{0}))^2 + O(\delta ^3), \end{aligned}$$

and so

$$\begin{aligned} \qquad \qquad \qquad \qquad \frac{1}{Z} = 1 - \frac{1}{2(k+2)}\,\sigma ^2 \sum _{i=1}^k H^i(\phi (\textbf{0}))^2 + O(\delta ^3).\qquad \qquad \qquad \qquad \end{aligned}$$

$$\square $$

### Proposed transport map

As mentioned in the introduction, when considering an embedded manifold, it is crucial to include the mean curvature in the transport map. We will show that the transport map proposed below is an approximate transport map with degree 3. We then present a criterion for optimality of the proposed map in Lemma 2.25.

#### Definition 2.18

Define $T :B_{\sigma ,\varepsilon }(x_0) \rightarrow B_{\sigma , \varepsilon }(y)$ in Fermi coordinates as

$$\begin{aligned} \begin{aligned} T(\phi (\alpha ,\beta )) :=\phi \biggl (&\delta -\alpha _1, \alpha _2, \ldots , \alpha _m, \\&\quad \beta _1-\frac{1}{2}\,(\sigma ^2 -\beta _1^2)(\delta -2\alpha _1)\hspace{0.55542pt}\partial _{\alpha _1} (H^1 \hspace{0.55542pt}{\circ }\hspace{1.111pt}\phi )(\textbf{0}),\\&\quad \ldots ,\\&\quad \beta _k-\frac{1}{2}\,(\sigma ^2 -\beta _k^2)(\delta -2\alpha _1)\hspace{0.55542pt}\partial _{\alpha _1} (H^k \hspace{0.55542pt}{\circ }\hspace{1.111pt}\phi )(\textbf{0})\biggr ). \end{aligned} \end{aligned}$$

Denote by $\hat{\alpha } = (\alpha _2,\ldots ,\alpha _m)$ and similarly for $\hat{\alpha }'$, and denote the input vector on the right in the above definition as . Note that

$$\begin{aligned} \alpha '_1 = \delta -\alpha _1, \quad \hat{\alpha }'=\hat{\alpha }, \quad \beta ' = \beta + O(\delta ^3). \end{aligned}$$

#### Remark 2.19

Observe that *T* is a local diffeomorphism and

$$\begin{aligned} \bigl |{\det D(\phi ^{-1} \hspace{0.55542pt}{\circ }\hspace{1.111pt}T \hspace{1.111pt}{\circ }\hspace{1.111pt}\phi )(\alpha ,\beta )}\bigr | = 1 - \sum _{i=1}^k \beta _i(\delta -2\alpha _1)\hspace{0.55542pt}\partial _{\alpha _1} (H^i\hspace{0.55542pt}{\circ }\hspace{1.111pt}\phi )(\textbf{0}) + O(\delta ^3) \end{aligned}$$

and deduce2.12

#### Remark 2.20

The third order terms in the definition of *T* are adjustments to cancel out second order terms in the proof of Proposition 2.23 below, obtaining an approximate transport of degree 3 as a result. In fact, the form of *T* is tailored precisely for this to occur. It turns out these third order adjustment terms do not influence the Wasserstein distance computation up to order 4.

We need two general lemmas to show that *T* is an approximate transport of degree 3.

#### Lemma 2.21

(Density under pushforward) Let  be measurable spaces,  a measurable bijection with measurable inverse, and $\mu , \nu$ two measures on  with $\mu \ll \nu$. Then the push-forward measures are also absolutely continuous with density

$$\begin{aligned} \frac{d(\psi _*\mu )}{d(\psi _*\nu )}\hspace{0.55542pt}(x) = \frac{d\mu }{d\nu }\hspace{0.55542pt}(\psi ^{-1}(x)). \end{aligned}$$

#### Proof

For all measurable sets ,

$$\begin{aligned} \qquad \qquad \qquad \qquad \qquad \psi _*\mu (A)&=\mu (\psi ^{-1}(A)) \\&=\int _{\psi ^{-1}(A)} \frac{d\mu }{d\nu }\hspace{0.55542pt}(x)\,d\nu (x)\\&= \int _{\psi ^{-1}(A)} \frac{d\mu }{d\nu }\hspace{0.55542pt}(\psi ^{-1}\hspace{0.55542pt}{\circ }\hspace{1.111pt}\psi (x))\,d\nu (x)\\&= \int _A \frac{d\mu }{d\nu }\hspace{0.55542pt}(\psi ^{-1}(x))\,d(\psi _*\nu )(x).\qquad \qquad \qquad \qquad \qquad \end{aligned}$$

$$\square $$

Noting that the representations (2.6) and (2.7) are decompositions into skew-products of two measures, the following will be used for density comparisons.

Let  be measurable spaces. Given measures  on  and a measure $\mu _2$ on , the skew-product is defined as follows: For all bounded measurable real valued functions *f* on ,

#### Lemma 2.22

(Skew-product density factorization) Consider two families of measures  and  on  such that $\mu _1^y \ll \nu _1^y$ for every  and the map $(x,y) \mapsto \frac{d\mu _1^y}{d\nu _1^y}(x)$ is measurable. Furthermore, let $\mu _2$ and $\nu _2$ be measures on  with $\mu _2 \ll \nu _2$. Consider the skew products of  with $\mu _2$ and that of  with $\nu _2$. Then $\mu _1 \hspace{0.55542pt}{\otimes }\hspace{1.111pt}\mu _2 \ll \nu _1 \hspace{0.55542pt}{\otimes }\hspace{1.111pt}\nu _2$ and

$$\begin{aligned} \frac{d(\mu _1\hspace{0.55542pt}{\otimes }\hspace{1.111pt}\mu _2)}{d(\nu _1 \hspace{0.55542pt}{\otimes }\hspace{1.111pt}\nu _2)}\hspace{0.55542pt}(x,y) = \frac{d\mu _1^y}{d\nu _1^y}\hspace{0.55542pt}(x) \, \frac{d\mu _1}{d\nu _2}\hspace{0.55542pt}(y). \end{aligned}$$

#### Proof

Plugging in the densities, for all  bounded:$\square$

We verify that the density of $T_*\mu _{x_0}^{\sigma ,\varepsilon }$ via *T* matches that of $\mu _y^{\sigma ,\varepsilon }$ up to $O(\delta ^3)$.

#### Proposition 2.23

The proposed map is an approximate transport map of degree 3, i.e.

$$\begin{aligned} \frac{d(T_* \mu _{x_0}^{\sigma ,\varepsilon })}{d\mu _y^{\sigma ,\varepsilon }}\hspace{0.55542pt}(\phi (\alpha ,\beta )) = 1 + O(\delta ^3). \end{aligned}$$

#### Proof

First, combining the elementary change of variable formula with the Fermi coordinate representation of $\mu _{x_0}^{\sigma ,\varepsilon }$, with notation of Definition 2.18 we have2.13using the expansions (2.12) for the determinant of $\phi ^{-1} \hspace{0.55542pt}{\circ }\hspace{1.111pt}T \hspace{1.111pt}{\circ }\hspace{1.111pt}\phi$ and (2.9) for the coordinate representation of $\phi ^{-1}_* \mu _{x_0}^{\sigma ,\varepsilon }$.

We use Lemma 2.21 to push the density into Fermi coordinates, and then Lemma 2.22 allows us to take the ratio of the densities of (2.13) and (2.9), obtaining

$$\begin{aligned} \begin{aligned} \frac{d(T_* \mu _{x_0}^{\sigma ,\varepsilon })}{d\mu _y^{\sigma ,\varepsilon }}\hspace{0.55542pt}(\phi (\alpha ,\beta ))&= \frac{d(\phi _*^{-1} T_* \mu _{x_0}^{\sigma ,\varepsilon })}{d(\phi _*^{-1} \mu _y^{\sigma ,\varepsilon })}\hspace{0.55542pt}(\alpha ,\beta ) \\&= \mathbb {1}_{\tilde{B}_{\sigma ,\varepsilon }(\textbf{0})}(\alpha _1-\delta , \hat{\alpha },\beta ) ( 1 + O(\delta ^3) ) \end{aligned} \end{aligned}$$

because the second order terms cancel out. Here we also used that *T* is a diffeomorphism from $\widetilde{B}_{\sigma , \varepsilon }(\textbf{0}_{m+k})$ to $\widetilde{B}_{\sigma , \varepsilon }(\delta , \textbf{0}_{m+k-1})$, hence$\square$

#### Remark 2.24

Building upon the preceding proposition and leveraging Corollary 2.13, we readily deduce that the proposed transport map satisfies:

$$\begin{aligned} W_1(\mu _{x_0}^{\sigma ,\varepsilon }, \mu _y^{\sigma ,\varepsilon }) = W_1(\mu _{x_0}^{\sigma ,\varepsilon }, T_* \mu _{x_0}^{\sigma ,\varepsilon }) + O(\delta ^4), \end{aligned}$$

taking also into account that $\text {supp}\, T_*\mu _{x_0}^{\sigma ,\varepsilon } = \text {supp}\, \mu _y^{\sigma ,\varepsilon }$ leading to $\text {diam}\, \text {supp}\, T_*\mu _{x_0}^{\sigma ,\varepsilon } = O(\delta )$ when $\sigma \vee \varepsilon \leqslant {\delta }/{4}$. Thus, when computing the coarse curvature, we may use $W_1(\mu _{x_0}^{\sigma ,\varepsilon }, T_* \mu _{x_0}^{\sigma ,\varepsilon })$. This is justified as terms involving the second fundamental form at the point $x_0$ emerge only at the third order in the expansion of $W_1(\mu _{x_0}^{\sigma ,\varepsilon }, \mu _y^{\sigma ,\varepsilon })$, making precision up to $O(\delta ^4)$ sufficient.

The following will allow us to deduce a Wasserstein lower bound from an upper bound provided by an approximate transport map of degree 3, and merging these into a both-sided estimate up to $O(\delta ^4)$.

#### Lemma 2.25

If  is smooth and takes the form

2.14 $$\begin{aligned} f(Tz) - f(z) = \Vert Tz - z\Vert + O(\delta ^4) = O(\delta ) \end{aligned}$$

and the magnitude of its gradient satisfiesthen for all $\sigma ,\varepsilon ,\delta$ sufficiently small with $\sigma \vee \varepsilon \leqslant {\delta }/{4}$,

$$\begin{aligned} W_1(\mu _{x_0}^{\sigma ,\varepsilon }, T_*\mu _{x_0}^{\sigma ,\varepsilon })&= \int \Vert Tz-z\Vert \, d\mu _{x_0}^{\sigma ,\varepsilon }(z) + O(\delta ^4) \\  &= \int (f(Tz) - f(z)) \, d\mu _{x_0}^{\sigma ,\varepsilon }(z) + O(\delta ^4). \end{aligned}$$

#### Proof

Using the expansion $$ {1}/({1+a}) = 1 - a + O(a^2)$$, we deduce thatBy the mean value theorem, a differentiable function divided by the supremum of its gradient is 1-Lipschitz. Then by Kantorovich–Rubinstein dualityusing the assumption (2.14) on the third and fourth line. $$\square $$

Finally, in order to integrate over the correct range of Fermi coordinates to cover precisely $B_{\sigma ,\varepsilon }(x_0)$ as the support of $\mu _{x_0}^{\sigma ,\varepsilon }$, we need to find the range parameter $\varepsilon (\alpha )$ such that if

$$\begin{aligned} \textbf{B}_{\sigma ,\varepsilon }(\textbf{0}) :=\biggl \{(\alpha , \beta ): |\alpha _1| \leqslant \varepsilon , \sum _{j=2}^m \alpha _j^2 \leqslant \varepsilon (\alpha )^2, \sum _{i=1}^k \beta _i^2 \leqslant \sigma ^2 \biggr \} \subset \mathbb {R}^{m+k} \end{aligned}$$

then

$$\begin{aligned} \phi (\textbf{B}_{\sigma , \varepsilon }(\textbf{0})) = B_\varepsilon (x_0). \end{aligned}$$

This is a necessary consideration, because in general non-flat spaces

$$\begin{aligned} \phi (\widetilde{B}_{\sigma ,\varepsilon }(\textbf{0})) \ne B_{\sigma ,\varepsilon }(x_0). \end{aligned}$$

The following is a classical result of Toponogov, which is a generalization of the Pythagoras theorem for Riemannian manifolds and gives a characterisation of sectional curvature. See e.g. [24] for a proof.

#### Lemma 2.26

For any point $x_0\in M$ and any $w_1,w_2 \in T_{x_0}M$ sufficiently small, the Riemannian distance between $\exp _{x_0} (w_1)$ and $\exp _{x_0} (w_2)$ has the expansion

$$\begin{aligned} d(\exp _{x_0}(w_1), \exp _{x_0}(w_2))= &   \Vert w_1-w_2\Vert - \frac{1}{3}\, \langle R(w_1,w_2)\hspace{0.55542pt}w_2,w_1 \rangle \\    &   \, + O(\max \hspace{0.55542pt}(\Vert w_1\Vert , \Vert w_2\Vert )^5). \end{aligned}$$

As a consequence, we deduce that given a coordinate $\alpha _1 \in (-\varepsilon , \varepsilon )$, the range parameter $\varepsilon (\alpha )$ is characterized by the relation

$$\begin{aligned} \varepsilon ^2 = \alpha _1^2 + \varepsilon (\alpha )^2 + O(\max \hspace{0.55542pt}(\alpha _1^2, \varepsilon (\alpha )^2)) \end{aligned}$$

where the coefficient in the remainder term only depends on a fixed neighbourhood of $x_0$. This implies $\varepsilon (\alpha ) = O(\varepsilon )$ and

$$\begin{aligned} \varepsilon (\alpha ) = (1+ O(\varepsilon ^2)) \sqrt{\varepsilon ^2 -\alpha _1^2} = \sqrt{\varepsilon ^2 -\alpha _1^2} + O(\varepsilon ^3). \end{aligned}$$

We shall label the remainder term $r(\alpha ) = O(\varepsilon ^3)$ for the purpose of the following proof. The next corollary will allow us to ignore the distinction between $\widetilde{B}_{\sigma ,\varepsilon }(\textbf{0})$ and $\textbf{B}_{\sigma ,\varepsilon }(\textbf{0})$ up to $O(\delta ^4)$ whenever we integrate with respect to the test measure $\mu _{x_0}^{\sigma ,\varepsilon }$ in Fermi coordinates.

#### Corollary 2.27

If  is a polynomial with no constant term and $\max \hspace{0.55542pt}(\sigma ,\varepsilon )\leqslant \delta$ then

#### Proof

We split the domain of integral on the right so that one part matches the domain on the left and the integral of the other part is $O(\delta ^4)$:on the last line we using that $P(\alpha ,\beta )$ has no constant term and $r(\alpha ) = O(\varepsilon ^3) = O(\delta ^3)$. $\square$

## Curves and surfaces

We establish explicit formulas for the coarse extrinsic curvature defined by (1.3) in four practically relevant cases: a circle, a planar curve, a space curve, and a surface. We begin by presenting the common setup shared among all these cases.

### The circle example

Our motivating example is the circle $S^1_R$ with a fixed radius $R>0$, which avoids technicalities arising from varying radius in the osculating circle, an issue that will be addressed in Sect. 3.2 in the case of planar curves.

#### Notation 3.1

Denote the polar coordinates

3.1 $$\begin{aligned} \phi (\alpha ,\beta ) :=\begin{pmatrix} (R-\beta ) \cos \hspace{0.55542pt}(\alpha /R)\\ (R-\beta ) \sin \hspace{0.55542pt}(\alpha /R) \end{pmatrix}, \end{aligned}$$

where $\alpha \in (-\pi R,\pi R)$ parametrizes arc-length distance from the point (*R*, 0) along the circle and $\beta \in (-\sigma , \sigma )$ parametrizes the direction normal to the circle.

Denote $x_0 :=\phi (0,0) = (R,0)$ and for every $\delta >0$ denote $$y :=\Bigl ({\begin{smallmatrix} R\cos \hspace{0.55542pt}(\delta /R)\\ R\sin \hspace{0.55542pt}(\delta /R)\end{smallmatrix}}\Bigr )$$.

#### Lemma 3.2

The test measures in polar coordinates take the form

$$\begin{aligned} \begin{aligned} (\phi _*^{-1} \mu _{y}^{\sigma , \varepsilon })(d\alpha ,d\beta )&= \frac{1}{4\sigma \varepsilon }\,\mathbb {1}_{(\delta -\varepsilon , \delta +\varepsilon )\times (-\sigma ,\sigma )}(\alpha , \beta )\biggl (1-\frac{\beta }{R}\biggr )\, d\alpha \, d\beta . \end{aligned} \end{aligned}$$

#### Proof

At any $(\alpha , \beta )$, the radial coordinate is $R-\beta$, the radial length element is $d\beta$ and the angular element is $\frac{d\alpha }{R}$, giving the volume element $(R-\beta ) \hspace{0.55542pt}d\beta \frac{d\alpha }{R} = (1-\frac{\beta }{R})\hspace{0.55542pt}d\alpha \hspace{0.55542pt}d\beta$, with $\frac{1}{4\sigma \varepsilon }$ as the probability normalization factor for the support $(\delta -\varepsilon , \delta +\varepsilon )\hspace{1.111pt}{\times }\hspace{1.111pt}(-\sigma ,\sigma )$.

This is consistent with the formula of Proposition 2.9, as the mean curvature at $(\alpha ,\beta )$ is $\frac{1}{R-\beta }$, which gives the density

$$\begin{aligned} e^{-\int _0^1 \frac{\beta }{R-s\beta }\hspace{0.55542pt}ds} = e^{\log \hspace{0.55542pt}(R-\beta ) - \log R} = 1-\frac{\beta }{R} \end{aligned}$$

on $$(\delta -\varepsilon , \delta +\varepsilon )\hspace{1.111pt}{\times }\hspace{1.111pt}(-\sigma ,\sigma )$$. $$\square $$

The transport map of Definition 2.18 boils down to

**Fig. 1:**
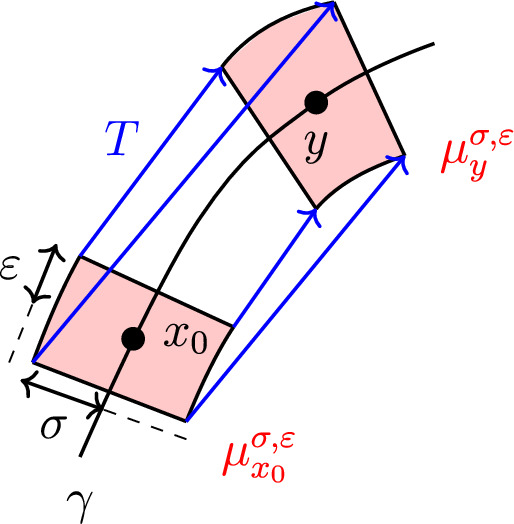
Planar curve case: test measures in red with some transport pairs of *T* in blue (Color figure online)

3.2 $$\begin{aligned} T(\phi (\alpha ,\beta )) = \phi (\delta -\alpha , \beta ) = \begin{pmatrix} (R-\beta ) \cos \hspace{0.55542pt}((\delta -\alpha )/R) \\ (R-\beta ) \sin \hspace{0.55542pt}((\delta -\alpha )/R)\end{pmatrix}, \end{aligned}$$

and note that $y = Tx_0 = T(\phi (0,0))$. See also Fig. 1 below.

#### Remark 3.3

In this case the transport map *T* is precise in the sense that $T_* \mu _{x_0}^{\sigma ,\varepsilon } = \mu _y^{\sigma , \varepsilon }$. Indeed, for any $f :\mathbb {R}^2 \rightarrow \mathbb {R}$ Borel measurable,

#### Proposition 3.4

For all $\delta , \varepsilon , \sigma >0$ sufficiently small with $\sigma \vee \varepsilon \leqslant {\delta }/{2}$, it holds that

$$\begin{aligned} \begin{aligned} W_1(\mu _{x_0}^{\sigma ,\varepsilon }, \mu _{y}^{\sigma ,\varepsilon })&=2R^2 \sin \hspace{0.55542pt}\biggl (\frac{\delta }{2R}\biggr )\,\frac{1}{\varepsilon }\sin \hspace{0.55542pt}\biggl (\frac{\varepsilon }{R}\biggr )\biggl (1+\frac{\sigma ^2}{3R^2}\biggr ) \\&= \Vert x_0-y\Vert \,\frac{R}{\varepsilon }\sin \hspace{0.55542pt}\biggl (\frac{\varepsilon }{R}\biggr ) \biggl (1+\frac{\sigma ^2}{3R^2}\biggr ). \end{aligned} \end{aligned}$$

#### Proof

For every point $z=\phi (\alpha ,\beta )$,

$$\begin{aligned} \Vert Tz-z\Vert = 2 (R-\beta ) \sin \hspace{0.55542pt}\biggl (\frac{\delta -2\alpha }{2R}\biggr ), \end{aligned}$$

which is the Euclidean distance of two points on the circle at angle $({\delta -2\alpha })/{R}$ apart. Integrating with respect to the test measure yields

$$\begin{aligned} \begin{aligned} W_1(\mu _{x_0}^{\sigma ,\varepsilon }, \mu _y^{\sigma ,\varepsilon })&\leqslant \int \Vert Tz-z\Vert \,d\mu _{x_0}^{\sigma ,\varepsilon }(z)\\&= \frac{1}{4\sigma \varepsilon } \int _{-\sigma }^\sigma d\beta \int _{-\varepsilon }^\varepsilon d\alpha \biggl (1-\frac{\beta }{R}\biggr )\hspace{0.55542pt}2 (R-\beta ) \sin \hspace{0.55542pt}\biggl (\frac{\delta -2\alpha }{2R}\biggr ) \\&= \frac{1}{4\sigma \varepsilon } \int _{-\sigma }^\sigma d\beta \biggl (1-\frac{\beta }{R}\biggr )\hspace{0.55542pt}2 (R-\beta ) \\&\qquad \hspace{1.111pt}{\times }\hspace{1.111pt}\int _{-\varepsilon }^\varepsilon d\alpha \biggl (\sin \hspace{0.55542pt}\biggl (\frac{\delta }{2R}\biggr ) \cos \hspace{0.55542pt}\biggl (\frac{\alpha }{R}\biggr ) - \sin \hspace{0.55542pt}\biggl (\frac{\alpha }{R}\biggr )\cos \hspace{0.55542pt}\biggl (\frac{\delta }{2R}\biggr )\biggr ) \\&= 2R^2 \sin \hspace{0.55542pt}\biggl (\frac{\delta }{2R}\biggr )\,\frac{1}{\varepsilon }\sin \hspace{0.55542pt}\biggl (\frac{\varepsilon }{R}\biggr )\biggl (1+\frac{\sigma ^2}{3R^2}\biggr ). \end{aligned} \end{aligned}$$

For the lower bound, we test against the 1-Lipschitz function

$$\begin{aligned} f(z) :=\biggl \langle z-x_0, \frac{y-x_0}{\Vert y-x_0\Vert } \biggr \rangle . \end{aligned}$$

We have

$$\begin{aligned} \begin{aligned} y-x_0 = \phi (\delta ,0)-\phi (0,0)&= \begin{pmatrix} R\cos \hspace{0.55542pt}(\delta /R) \\ R\sin \hspace{0.55542pt}(\delta /R) \end{pmatrix} -\begin{pmatrix} R \\ 0 \end{pmatrix} = \begin{pmatrix} R(\cos \hspace{0.55542pt}(\delta /R)-1)\\ R\sin \hspace{0.55542pt}(\delta /R)\end{pmatrix}, \end{aligned} \end{aligned}$$

and so $\Vert y-x_0\Vert = R\sqrt{2(1-\cos \hspace{0.55542pt}(\delta /R)} = 2R \sin \hspace{0.55542pt}(\delta /(2R))$, giving

3.3 $$\begin{aligned} \frac{y-x_0}{\Vert y-x_0\Vert } = \frac{1}{2 \sin \hspace{0.55542pt}(\delta /(2R))} \begin{pmatrix} \cos \hspace{0.55542pt}(\delta / R)-1 \\ \sin \hspace{0.55542pt}(\delta /R)\end{pmatrix}. \end{aligned}$$

Then we compute using (3.1), (3.2) and (3.3):

$$\begin{aligned} \begin{aligned} f(Tz)-f(z)&= \biggl \langle \phi (\delta -\alpha ,\beta ) - \phi (\alpha ,\beta ), \frac{y-x_0}{\Vert y-x_0\Vert } \biggr \rangle \\&= \frac{(R-\beta )}{2 \sin \hspace{0.55542pt}(\delta /(2R))} \begin{pmatrix}\cos \hspace{0.55542pt}((\delta -\alpha )/R) - \cos \hspace{0.55542pt}(\alpha /R) \\ \sin \hspace{0.55542pt}((\delta -\alpha )/R) - \sin \hspace{0.55542pt}(\alpha /R) \end{pmatrix} \hspace{0.55542pt}{\cdot }\hspace{1.111pt}\begin{pmatrix} \cos \hspace{0.55542pt}(\delta /R)-1\\ \sin \hspace{0.55542pt}(\delta /R)\end{pmatrix} \\&= \frac{R-\beta }{\sin \hspace{0.55542pt}(\delta /(2R))}\, \bigl (\cos \hspace{0.55542pt}(\alpha /R) - \cos \hspace{0.55542pt}((\delta -\alpha )/R) \bigr )\\&= 2(R-\beta ) \sin \hspace{0.55542pt}((\delta -2\alpha )/(2R)) = \Vert Tz-z\Vert \end{aligned} \end{aligned}$$

by trigonometric identities. Therefore

$$\begin{aligned} \begin{aligned} W_1(\mu _{x_0}^{\sigma ,\varepsilon }, \mu _y^{\sigma ,\varepsilon })&\geqslant \int f(z)\hspace{0.55542pt}(d\mu ^{\sigma ,\varepsilon }_{y}(z)-d\mu ^{\sigma ,\varepsilon }_{x_0}(z)) \\&= \int (f(Tz) -f(z))\, d\mu ^{\sigma ,\varepsilon }_{x_0}(z) \\&= \int \Vert Tz-z\Vert \,d\mu ^{\sigma ,\varepsilon }_{x_0}(z), \end{aligned} \end{aligned}$$

which shows the lower bound agrees exactly with the upper bound. $\square$

### Planar curve

Let $\gamma :(-\delta _0,\delta _0) \rightarrow \mathbb {R}^2$ be a smooth unit speed curve. As before, let $x_0:=\gamma (0)$, $y :=\gamma (\delta )$ where $\delta \in (-\delta _0, \delta _0)$.

The normal vector field along $\gamma$ is given by $\textbf{n}(\alpha ) :=\frac{\ddot{\gamma }(\alpha )}{\Vert \ddot{\gamma }(\alpha ) \Vert }$, the radius of the osculating circle is $R(\alpha ) :=\frac{1}{\Vert \ddot{\gamma }(\alpha )\Vert }$ and we have the relationships

3.4 $$\begin{aligned} \begin{aligned} \ddot{\gamma }(\alpha )&= \frac{\textbf{n}(\alpha )}{R(\alpha )}\hspace{0.55542pt},  &   \dddot{\gamma }(\alpha )= -\frac{1}{R(\alpha )^2}\, \dot{\gamma }(\alpha )- \frac{\dot{R}(\alpha )}{R(\alpha )^2}\,\textbf{n}(\alpha ),\\ \dot{\textbf{n}}(\alpha )&=  -\frac{\dot{\gamma }(\alpha )}{R(\alpha )}\hspace{0.55542pt},  &   \ddot{\textbf{n}}(\alpha ) = \frac{\dot{R}(\alpha )}{R(\alpha )^2}\,\dot{\gamma }(\alpha ) - \frac{1}{R(\alpha )^2}\, \textbf{n}(\alpha ). \end{aligned} \end{aligned}$$

Let $\phi :(-\delta _0,\delta _0) \hspace{1.111pt}{\times }\hspace{1.111pt}(-\sigma _0,\sigma _0) \rightarrow \mathbb {R}^2$ be given as follows:

$$\begin{aligned} \phi (\alpha ,\beta ) :=\gamma (\alpha )+\beta \textbf{n}(\alpha ). \end{aligned}$$

This is the Fermi chart along $\gamma$. While we have the general Fermi coordinate representation in terms of the expansion in Lemma 2.17, in this case we arrive at a precise form:

#### Lemma 3.5

The test measures at $y = \gamma (\delta )$ are

$$\begin{aligned} \begin{aligned} (\phi _*^{-1} \mu _{y}^{\sigma , \varepsilon })(d\alpha ,d\beta )&= \frac{1}{4\sigma \varepsilon }\,\mathbb {1}_{(\delta -\varepsilon , \delta +\varepsilon )\hspace{1.111pt}{\times }\hspace{1.111pt}(-\sigma ,\sigma )}(\alpha , \beta )\biggl (1-\frac{\beta }{R(\alpha )} \biggr )\, d\alpha \, d\beta . \end{aligned} \end{aligned}$$

#### Proof

To evaluate $H(\phi (\alpha ,\beta ))$ in applying Proposition 2.9, normalize the vector field tangent to the curve $\alpha \mapsto \phi (\alpha ,\beta )$ and compute the second derivative in $\mathbb {R}^2$ as

$$\begin{aligned} \begin{aligned} \frac{\partial _\alpha }{\Vert \partial _\alpha \phi (\alpha ,\beta ) \Vert }\,\biggl ( \frac{\partial _\alpha \phi (\alpha ,\beta )}{\Vert \partial _\alpha \phi (\alpha ,\beta )\Vert } \biggr ) = \frac{\partial _\alpha ^2 \phi (\alpha ,\beta )}{\Vert \partial _\alpha \phi (\alpha ,\beta )\Vert ^2} - \frac{\langle \partial _\alpha ^2 \phi (\alpha ,\beta ), \partial _\alpha \phi (\alpha ,\beta )\rangle }{ \Vert \partial _\alpha \phi (\alpha ,\beta )\Vert ^3 }\, \partial _\alpha \phi (\alpha ,\beta ). \end{aligned} \end{aligned}$$

The second term is tangential to the curve, so may be ignored for the computation of *H*. Moreover,Note that $\textbf{n}(\alpha )$ is normal to $\alpha \mapsto \phi (\alpha ,\beta )$ for every $\beta$ since

$$\begin{aligned} \langle \textbf{n}(\alpha ), \partial _\alpha \phi (\alpha ,\beta )\rangle =\langle \textbf{n}(\alpha ), \dot{\gamma }(\alpha ) + \beta \dot{\textbf{n}}(\alpha )\rangle = 0, \end{aligned}$$

therefore the mean curvature isFinally,and the Lebesgue measure of the support $\frac{1}{4\sigma \varepsilon }$ is the normalization factor because the $\beta$ term vanishes when integrating over $\beta \in (-\sigma ,\sigma )$. $\square$

In this case the proposed transport map of Definition 2.18 reduces to

$$\begin{aligned} T(\phi (\alpha , \beta )) = \phi \biggl (\delta -\alpha , \beta - \frac{1}{2}\,\frac{\dot{R}(0)}{R(0)^2}\,(\sigma ^2-\beta ^2)(\delta -2\alpha )\biggr ). \end{aligned}$$

As a consequence of Corollary 2.13,

#### Lemma 3.6

For all $\delta , \varepsilon , \sigma >0$ sufficiently small with $\sigma \vee \varepsilon \leqslant {\delta }/{4}$, it holds that

$$\begin{aligned} W_1(\mu _{x_0}^{\sigma ,\varepsilon }, \mu _y^{\sigma ,\varepsilon }) = W_1(\mu ^{\sigma ,\varepsilon }_{x_0}, T_* \mu ^{\sigma ,\varepsilon }_{x_0}) + O(\delta ^4). \end{aligned}$$

For notational ease we shall from here onwards denote $R:=R(0)$ and $\dot{R}:=\dot{R}(0)$.

#### Proposition 3.7

Let $\gamma$ be a smooth unit speed curve in $\mathbb {R}^2$ such that $\gamma (0) = x_0$ and $\gamma (\delta )=y$. For all $\delta , \varepsilon , \sigma >0$ sufficiently small with $\sigma \vee \varepsilon \leqslant {\delta }/{4}$, it holds that

$$\begin{aligned} \begin{aligned} W_1(\mu _{x_0}^{\sigma ,\varepsilon }, \mu _{y}^{\sigma ,\varepsilon })&= \Vert x_0-y\Vert \biggl ( 1-\frac{\varepsilon ^2}{6R^2} + \frac{\sigma ^2}{3R^2} \biggr ) + O(\delta ^4) \end{aligned} \end{aligned}$$

where *R* is the radius of the osculating circle of the curve at $x_0$.

#### Proof

Lemma 3.6 allows computing $W_1(\mu ^{\sigma ,\varepsilon }_{x_0}, T_* \mu ^{\sigma ,\varepsilon }_{x_0})$ instead. Throughout the proof, terms of order $\delta ^4$ and higher are absorbed into $O(\delta ^4)$. For the upper bound, we compute by expansion with respect to the orthonormal basis $(\dot{\gamma }(0), \textbf{n}(0))$ at $x_0$,

$$\begin{aligned} \begin{aligned} \phi (\alpha , \beta )&= \gamma (0) + \beta \textbf{n}(0) + \alpha (\dot{\gamma }(0) + \beta \dot{\textbf{n}}(0)) \\&\qquad \qquad \qquad + \frac{\alpha ^2}{2}\, (\ddot{\gamma }(0) + \beta \ddot{\textbf{n}}(0)) + \frac{\alpha ^3}{6} \,\dddot{\gamma }(0) + O(\delta ^4) \\&= x_0 + \biggl ( \alpha - \frac{\alpha \beta }{R} - \frac{\alpha ^3}{6R^2} + \frac{\beta \alpha ^2 \dot{R}}{2R^2} \biggr )\hspace{0.55542pt}\dot{\gamma }(0) \\&\qquad \quad + \biggl (\beta + \frac{\alpha ^2}{2R} - \frac{\alpha ^3 \dot{R}}{6R^2} - \frac{\beta \alpha ^2}{2R^2} \biggr )\hspace{0.55542pt}\textbf{n}(0) + O(\delta ^4) \end{aligned} \end{aligned}$$

having inserted for the derivatives at 0 using the list (3.4). Then the distance of the transport pairs up to order 4 is

3.5 $$\begin{aligned} \begin{aligned}&\Vert T(\phi (\alpha , \beta ))-\phi (\alpha ,\beta )\Vert = \Vert \phi (\delta -\alpha , \beta ) - \phi (\alpha ,\beta )\Vert \\&\;\;=(\delta -2\alpha )\biggl \Vert \biggl (1 - \frac{\beta }{R}-\frac{1}{6R^2} \,(\delta ^2-\delta \alpha +\alpha ^2) +\frac{\beta \dot{R}}{2R^2}\,\delta + O(\delta ^3) \biggr )\hspace{0.55542pt}\dot{\gamma }(0) \\&\qquad \qquad \qquad \qquad \qquad \qquad \qquad \qquad \qquad \qquad \qquad \qquad + \biggl (\frac{\delta }{2R} + O(\delta ^2)\biggr )\hspace{0.55542pt}\textbf{n}(0)\biggr \Vert \end{aligned} \end{aligned}$$

having used the factorizations $(\delta -\alpha )^3-\alpha ^3 = (\delta -2\alpha )(\delta ^2-\delta \alpha +\alpha ^2)$ and $(\delta -\alpha )^2 - \alpha ^2 = \delta (\delta -\alpha )$. By orthonormality of $(\dot{\gamma }(0), \textbf{n}(0))$, we compute this norm as3.6by the expansion $\sqrt{1+x} = 1+\frac{1}{2}x-\frac{1}{8}x^2+O(x^3)$ for the square root on the last line.

Moreover, expanding the volume distortion factor as

$$\begin{aligned} \biggl (1-\frac{\beta }{R(\alpha )}\biggr ) = 1- \frac{\beta }{R} + \frac{\dot{R}}{R^2}\,\alpha \beta + O(\delta ^3) \end{aligned}$$

and multiplying the expression for $\Vert T(\phi (\alpha , \beta ))-\phi (\alpha ,\beta )\Vert$ by this factor, we integrate and note that only terms of even order in both $\alpha$ and $\beta$ contribute, yielding

$$\begin{aligned} \begin{aligned}&W_1(\mu _{x_0}^{\sigma ,\varepsilon }, \mu _y^{\sigma , \varepsilon }) \\&\;\;\leqslant \frac{1}{4\sigma \varepsilon } \int _{-\varepsilon }^\varepsilon d\alpha \int _{-\sigma }^\sigma d\beta \biggl (1- \frac{\beta }{R}+\frac{\dot{R}}{R^2}\,\alpha \beta +O(\delta ^3)\biggr ) \Vert T(\phi (\alpha , \beta ))-\phi (\alpha ,\beta )\Vert \\&\;\;= \frac{1}{4\sigma \varepsilon } \int _{-\varepsilon }^\varepsilon d\alpha \int _{-\sigma }^\sigma d\beta \biggl (1- \frac{\beta }{R}+\frac{\dot{R}}{R^2}\,\alpha \beta +O(\delta ^3)\biggr )\\&\quad \qquad \hspace{1.111pt}{\times }\hspace{1.111pt}(\delta -2\alpha ) \biggl ( 1 -\frac{\beta }{R} - \frac{\delta ^2}{24R^2} + \frac{\delta \alpha }{6R^2} - \frac{\alpha ^2}{6R^2}+ \frac{ \dot{R}}{2R^2}\, \beta \delta \biggr ) + O(\delta ^4)\\&\;\;=\delta \biggl (1 - \frac{\delta ^2}{24R^2} -\frac{\varepsilon ^2}{6R^2} + \frac{\sigma ^2}{3R^2} \biggr ) + O(\delta ^4) \\&\;\;= \Vert y-x_0\Vert \biggl ( 1-\frac{\varepsilon ^2}{6R^2} + \frac{\sigma ^2}{3R^2} \biggr ) + O(\delta ^4). \end{aligned} \end{aligned}$$

To obtain the factor $\Vert y-x_0\Vert$ on the last line, we applied that

$$\begin{aligned} \Vert y-x_0\Vert = \delta \biggl (1-\frac{\delta ^2}{24R^2}\biggr )+O(\delta ^4) \end{aligned}$$

which can be deduced by plugging in for $\alpha =\beta =0$ in the previous computation of $T(\phi (\alpha ,\beta ))-\phi (\alpha ,\beta )$. The $\sigma ^2$ coefficient came from integrating the $\beta ^2$ term of the integrand, $\frac{\sigma ^2}{3R^2} = \frac{1}{2\sigma }\int _{-\sigma }^\sigma \bigl (-\frac{\beta }{R}\bigr ) \hspace{1.111pt}{\times }\hspace{1.111pt}\bigl (-\frac{\beta }{R}\bigr )\hspace{0.55542pt}d\beta$. The terms with odd power in $\alpha$ or $\beta$ such as $\delta \alpha \beta$ vanished as they are mean zero.

We proceed with showing the lower bound, using again the 1-Lipschitz test function

$$\begin{aligned} f(z) :=\biggl \langle z-x_0, \frac{y-x_0}{\Vert y-x_0\Vert } \biggr \rangle . \end{aligned}$$

Express the vector between the centres of the two test measures, recalling $\gamma (0)=x_0$,

$$\begin{aligned} \begin{aligned} \gamma (\delta )-\gamma (0)&= T(\phi (0,0)) - \phi (0,0) \\&= \delta \biggl (1- \frac{\delta ^2}{6R^2}\biggr )\hspace{0.55542pt}\dot{\gamma }(0) - \delta \biggl (\frac{\delta }{2R} + O(\delta ^2) \biggr )\hspace{0.55542pt}\textbf{n}(0) + O(\delta ^4). \end{aligned} \end{aligned}$$

This vector has magnitude

$$\begin{aligned} \Vert \gamma (\delta )-\gamma (0)\Vert = \delta \biggl ( 1-\frac{\delta ^2}{24R^2} \biggr ) +O(\delta ^4), \end{aligned}$$

and so we deduce that

$$\begin{aligned} \begin{aligned} \frac{y-x_0}{\Vert y-x_0\Vert }&= \frac{\gamma (\delta )-\gamma (0)}{\Vert \gamma (\delta )-\gamma (0)\Vert } = \biggl (1-\frac{\delta ^2}{8R^2} + O(\delta ^3)\biggr )\hspace{0.55542pt}\dot{\gamma }(0) + \biggl (\frac{\delta }{2R} + O(\delta ^2) \biggr )\hspace{0.55542pt}\textbf{n}(0). \end{aligned} \end{aligned}$$

Then we compute, using the expression (3.5) for $T(\phi (\alpha ,\beta )) - \phi (\alpha ,\beta )$ obtained above,

$$\begin{aligned} \begin{aligned}&f(Tz)-f(z)\\&\;\;= \biggl \langle T(\phi (\alpha ,\beta )) - \phi (\alpha ,\beta ), \frac{y-x_0}{\Vert y-x_0\Vert } \biggr \rangle \\&\;\;= (\delta -2\alpha )\biggl (1 - \frac{\beta }{R}-\frac{1}{6R^2}\, (\delta ^2-\delta \alpha +\alpha ^2) +\frac{\beta \delta \dot{R}}{2R^2}\biggr ) \biggl (1-\frac{\delta ^2}{8R^2}+O(\delta ^3)\biggr ) \\&\qquad \qquad + (\delta -2\alpha ) \biggl (\frac{\delta }{2R} + O(\delta ^2)\biggr )\biggl (\frac{\delta }{2R} +O(\delta ^2)\biggr ) +O(\delta ^4)\\&\;\;= (\delta -2\alpha ) \biggl (1 - \frac{\beta }{R} -\frac{\delta ^2}{24R^2} +\frac{\delta \alpha }{6R^2} -\frac{\alpha ^2}{6R^2} +\frac{\beta \delta \dot{R}}{2R^2}\biggr ) + O(\delta ^4). \end{aligned} \end{aligned}$$

We see that this agrees with the pairwise transport distance (3.6) up to $O(\delta ^4)$, hence Lemma 2.25 applies and the upper and lower bounds agree up to an $O(\delta ^4)$ term. $\square$

### Space curve

Let $\gamma :(-\delta _0,\delta _0) \rightarrow \mathbb {R}^3$ be a smooth, unit speed curve with velocity $\dot{\gamma }$. Define the unit normal and binormal vector fields along $\gamma$ as

$$\begin{aligned} \textbf{n}(\alpha ) :=\frac{\ddot{\gamma }(\alpha )}{\Vert \ddot{\gamma }(\alpha ) \Vert }\hspace{0.55542pt}, \quad \textbf{b}(\alpha ) :=\frac{\dot{\gamma }(\alpha ) \hspace{1.111pt}{\times }\hspace{1.111pt}\textbf{n}(\alpha )}{\Vert \dot{\gamma }(\alpha ) \hspace{1.111pt}{\times }\hspace{1.111pt}\textbf{n}(\alpha )\Vert }\hspace{0.55542pt}. \end{aligned}$$

This yields the so-called Frenet–Serret frame $(\dot{\gamma }(\alpha ), \textbf{n}(\alpha ), \textbf{b}(\alpha ))$ of $\mathbb {R}^3$ along $\gamma$. Writing $R(\alpha ):=\frac{1}{\Vert \ddot{\gamma }(\alpha )\Vert }$ for the radius of the osculating circle and $\tau (\alpha ):=\Vert \dot{\textbf{b}}(\alpha )\Vert$ for the torsion, the Frenet–Serret formulas give relationships between the vector fields of the frame,

3.7 $$\begin{aligned} \begin{aligned} \ddot{\gamma }(\alpha )&= \frac{\textbf{n}(\alpha )}{R(\alpha )}\hspace{0.55542pt}, \\ \dot{\textbf{n}}(\alpha )&=  -\frac{\dot{\gamma }(\alpha )}{R(\alpha )} + \tau (\alpha )\hspace{0.55542pt}\textbf{b}(\alpha ), \\ \dot{\textbf{b}}(\alpha )&=  -\tau (\alpha )\hspace{0.55542pt}\textbf{n}(\alpha ). \end{aligned} \end{aligned}$$

From these, we deduce the higher order derivatives

3.8 $$\begin{aligned} \begin{aligned} \dddot{\gamma }(\alpha )&=  -\frac{1}{R(\alpha )^2}\, \dot{\gamma }(\alpha ) - \frac{\dot{R}(\alpha )}{R(\alpha )^2}\,\textbf{n}(\alpha ) + \frac{\tau (\alpha )}{R(\alpha )}\, \textbf{b}(\alpha ),\\ \ddot{\textbf{n}}(\alpha )&= \frac{\dot{R}(\alpha )}{R(\alpha )^2}\,\dot{\gamma }(\alpha ) - \biggl (\tau (\alpha )^2+\frac{1}{R(\alpha )^2}\biggr )\hspace{0.55542pt}\textbf{n}(\alpha ) + \dot{\tau }(\alpha )\hspace{0.55542pt}\textbf{b}(\alpha ),\\ \ddot{\textbf{b}}(\alpha )&= \frac{\tau (\alpha )}{R(\alpha )}\,\dot{\gamma }(\alpha ) -\dot{\tau }(\alpha )\hspace{0.55542pt}\textbf{n}(\alpha )-\tau (\alpha )^2 \hspace{1.111pt}\textbf{b}(\alpha ). \end{aligned} \end{aligned}$$

We will employ the Frenet–Serret frame for explicit computations of distances between points in the tubular neighborhood of a space curve. Additionally, we will employ it in formulating a sufficiently accurate approximate transport map between test measures, represented through an expansion in Fermi coordinates.

Definition 2.14 for Fermi coordinates requires a choice of a local orthonormal frame of the normal bundle along $\gamma$. We choose $(\textbf{n}_1,\textbf{n}_2)$ as follows:

$$\begin{aligned} \begin{aligned} \textbf{n}_1(\alpha )&:=\frac{\textbf{n}(\alpha ) - \alpha \tau (\alpha )\hspace{0.55542pt}\textbf{b}(\alpha )}{\sqrt{1+\alpha ^2\tau (\alpha )^2}} = (1+O(\alpha ^2))\hspace{0.55542pt}\textbf{n}(\alpha ) - (\alpha \tau (\alpha )+O(\alpha ^2))\hspace{0.55542pt}\textbf{b}(\alpha )),\\ \textbf{n}_2(\alpha )&:=\frac{\textbf{b}(\alpha ) + \alpha \tau (\alpha )\hspace{0.55542pt}\textbf{n}(\alpha )}{\sqrt{1+\alpha ^2\tau (\alpha )^2}} = (1+O(\alpha ^2))\hspace{0.55542pt}\textbf{b}(\alpha ) + (\alpha \tau (\alpha )+O(\alpha ^2))\hspace{0.55542pt}\textbf{n}(\alpha ) \end{aligned} \end{aligned}$$

where $\textbf{n}, \textbf{b}$ come from the Frenet–Serret frame.

#### Definition 3.8

Define the Fermi coordinates $\phi :(-\delta _0,\delta _0)^3 \rightarrow \mathbb {R}^3$, adapted to $\gamma$, by the formula:

$$\begin{aligned} \begin{aligned}&\phi (\alpha , \beta _1, \beta _2) :=\gamma (\alpha ) +\beta _1 \textbf{n}_1(\alpha ) + \beta _2 \textbf{n}_2(\alpha )\\&\qquad = \gamma (\alpha ) + (\beta _1 + \alpha \beta _2 \tau (\alpha )+O(\delta ^3)) \hspace{0.55542pt}\textbf{n}(\alpha ) + (\beta _2 - \alpha \beta _1 \tau (\alpha )+O(\delta ^3))\hspace{0.55542pt}\textbf{b}(\alpha ). \end{aligned} \end{aligned}$$

Denote $$R=R(0)$$, $$\dot{R}=\dot{R}(0)$$, $$\tau =\tau (0)$$, $$\dot{\tau }=\dot{\tau }(0)$$.

Consider the family of curves $\{\alpha \mapsto \phi (\alpha ,\beta _1,\beta _2) \,{:}\, (\beta _1,\beta _2) \in B_\sigma \}.$ Denote the particular unit normal vector fields

$$\begin{aligned} \tilde{\textbf{n}}(\alpha , \beta _1,\beta _2) :=\frac{1}{\sqrt{\beta _1^2+\beta _2^2}} \,(\beta _1 \textbf{n}_1(\alpha ) + \beta _2 \textbf{n}_2(\alpha ) ). \end{aligned}$$

The mean curvature of each curve in the direction $\tilde{\textbf{n}}$ is expressed as

$$\begin{aligned} \begin{aligned}&\bigl \langle H(\phi (\alpha , \beta _1,\beta _2)), \tilde{\textbf{n}}(\alpha ,\beta _1,\beta _2)\bigr \rangle \\&\qquad = \biggl \langle \tilde{\textbf{n}}(\alpha , \beta _1,\beta _2), \frac{\partial _\alpha }{\Vert \partial _\alpha \phi (\alpha ,\beta _1,\beta _2)\Vert } \biggl ( \frac{\partial _\alpha \phi (\alpha ,\beta _1,\beta _2)}{\Vert \partial _\alpha \phi (\alpha ,\beta _1,\beta _2)\Vert } \biggr )\biggr \rangle \\ \\  &\qquad = \biggl \langle \tilde{\textbf{n}}(\alpha , \beta _1,\beta _2), \frac{\partial _\alpha ^2 \phi (\alpha ,\beta _1,\beta _2)}{\Vert \partial _\alpha \phi (\alpha ,\beta _1,\beta _2)\Vert ^2} \biggr \rangle . \end{aligned} \end{aligned}$$

where the second equality holds because $\tilde{\textbf{n}}$ is normal to $\partial _\alpha \phi$ by Lemma 2.4.

#### Remark 3.9

We perform computations in terms of the Frenet–Serret frame as it can be interpreted in terms of the radius of the osculating circle and torsion of the curve.

**Fig. 2:**
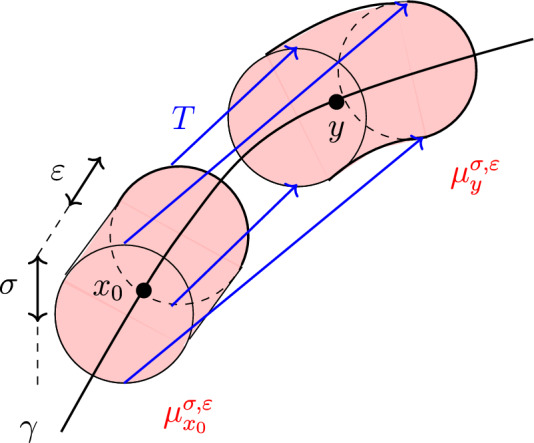
Space curve case: test measures in red with some transport pairs of *T* in blue (Color figure online)

1. As a special case of Lemma 2.17, using that the mean curvature components are
  $$\begin{aligned} \begin{aligned} H^1(\phi (\textbf{0}))&= \langle \textbf{n}(0), \ddot{\gamma }(0)\rangle = \biggl \langle \textbf{n}(0), \frac{\textbf{n}(0)}{R(0)}\biggr \rangle = \frac{1}{R(0)}\hspace{0.55542pt},\\ H^2(\phi (\textbf{0}))&= \langle \textbf{b}(0), \ddot{\gamma }(0)\rangle = \biggl \langle \textbf{b}(0), \frac{\textbf{n}(0)}{R(0)}\biggr \rangle = 0, \end{aligned} \end{aligned}$$
  the test measures at every $y = \gamma (\delta )$ in Fermi coordinates along $\gamma$ are
  $$\begin{aligned} \begin{aligned}&(\phi ^{-1}_* \mu _y^{\sigma ,\varepsilon })(d\alpha ,d\beta _1,d\beta _2) \\&\qquad = \frac{\mathbb {1}_{\tilde{B}_{\sigma ,\varepsilon }}(\alpha ,\beta _1,\beta _2) }{\int _{\tilde{B}_{\sigma ,\varepsilon }} (1+ r(\alpha ,\beta _1,\beta _2))\, d (\phi ^{-1}_* \mu _y^{\sigma ,\varepsilon })(\alpha ,\beta _1,\beta _2)} \\&\qquad \qquad \qquad \times \biggl (1- \frac{\beta _1}{R(0)} + r(\alpha ,\beta _1,\beta _2)\biggr )\, d\alpha \,d\beta _1\, d\beta _2 \end{aligned} \end{aligned}$$
  where $r(\alpha ,\beta _1,\beta _2) = O(\delta ^2)$ is the remainder.
2. The proposed transport map of Definition 2.18 reduces, in this case, to
  $$\begin{aligned} \begin{aligned} T(\phi (\alpha ,\beta _1,\beta _2)) = \phi \bigl (\delta -\alpha , \beta _1 + O(\delta ^3), \beta _2 + O(\delta ^3)\bigr ). \end{aligned} \end{aligned}$$
  The transport map in this case is depicted in Fig. 2. These expressions will be used in the proof of the next theorem.

#### Theorem 3.10

Let $\gamma :(-\delta _0,\delta _0) \rightarrow \mathbb {R}^3$ be a space curve with $x_0 = \gamma (0), y=\gamma (\delta )$ and $\mu _{x_0}^{\sigma ,\varepsilon }, \mu _y^{\sigma ,\varepsilon }$ the test measures defined in Definition 2.16 with coordinate representation of Remark 3.9. For all $\delta , \sigma , \varepsilon >0$ sufficiently small and with $\sigma \vee \varepsilon \leqslant {\delta }/{4}$, it holds that

$$\begin{aligned} \begin{aligned} W_1(\mu _{x_0}^{\sigma ,\varepsilon }, \mu _{y}^{\sigma ,\varepsilon })&=\Vert x_0-y\Vert \biggl (1 + \frac{\sigma ^2}{4R^2} - \frac{\varepsilon ^2}{6R^2} \biggr ) +O(\delta ^4). \end{aligned} \end{aligned}$$

where $R=\frac{1}{\Vert \ddot{\gamma }(0)\Vert }$ is the radius of the osculating circle.

#### Proof

Due to Lemma 3.6, it is sufficient to work with the distance $W_1(\mu _{x_0}^{\sigma ,\varepsilon }, T_* \mu _{x_0}^{\sigma ,\varepsilon })$ as it approximates $W_1(\mu _{x_0}^{\sigma ,\varepsilon }, \mu _{y}^{\sigma ,\varepsilon })$. The computation of the pairwise distances is similar to the planar curve case, (3.5), with additional terms due to the component $\textbf{b}(\alpha )$. Concretely, since

$$\begin{aligned} \begin{aligned} \textbf{b}(\alpha )&= \textbf{b}(0) + \alpha \dot{\textbf{b}}(0) + \frac{1}{2}\,\alpha ^2 \ddot{\textbf{b}}(0) + O(\alpha ^3) \\&= \frac{\alpha ^2 \tau }{2R}\, \dot{\gamma }(0) - \biggl (\alpha \tau + \frac{1}{2}\,\alpha ^2 \dot{\tau }\biggr )\hspace{0.55542pt}\textbf{n}(0) + \biggl (1 - \frac{1}{2}\,\alpha ^2 \tau ^2 \biggr )\hspace{0.55542pt}\textbf{b}(0) + O(\alpha ^3), \end{aligned} \end{aligned}$$

and using the derivatives (3.7) and (3.8), compute

$$\begin{aligned} \phi (\alpha ,\beta _1,\beta _2) = x_0&+ \biggl ( \alpha - \frac{\alpha \beta _1}{R} - \frac{\alpha ^3}{6R^2} + \frac{\beta _1 \alpha ^2 \dot{R}}{2R^2} + \frac{\beta _2 \alpha ^2 \tau }{2R} + O(\delta ^4) \biggr ) \hspace{0.55542pt}\dot{\gamma }(0) \\&+ \biggl (\beta _1 + \frac{\alpha ^2}{2R} + O(\delta ^3) \biggr ) \hspace{0.55542pt}\textbf{n}(0) + ( \beta _2 + O(\delta ^3) ) \hspace{0.55542pt}\textbf{b}(0). \end{aligned}$$

Then similarly to (3.5) we obtain

3.9 $$\begin{aligned} \begin{aligned}&\Vert T(\phi (\alpha , \beta _1,\beta _2))-\phi (\alpha ,\beta _1,\beta _2)\Vert \\&\quad = \bigl \Vert \gamma (\delta -\alpha ) + \beta _1 \textbf{n}(\delta -\alpha ) + \beta _2 \textbf{b}(\delta -\alpha ) -\gamma (\alpha )-\beta _1 \textbf{n}(\alpha ) - \beta _2 \textbf{b}(\alpha ) \bigr \Vert \\&\quad =(\delta -2\alpha ) \biggl \Vert \biggl (1 - \frac{\beta _1}{R}-\frac{1}{6R^2}\, (\delta ^2-\delta \alpha +\alpha ^2) +\frac{\beta _1 \dot{R}}{2R^2}\delta + \frac{\beta _2 \delta \tau }{2R} + O(\delta ^3) \biggr ) \dot{\gamma }(0)\\&\qquad \qquad \qquad \quad + \biggl (\frac{1}{2R} \,\delta + O(\delta ^2) \biggr ) \hspace{0.55542pt}\textbf{n}(0) +O(\delta ^2)\hspace{0.55542pt}\textbf{b}(0) \biggr \Vert \\&\quad = (\delta -2\alpha ) \biggl ( 1 -\frac{\beta _1}{R} - \frac{\delta ^2}{24R^2} + \frac{\delta \alpha }{6R^2} - \frac{\alpha ^2}{6R^2} + \frac{\beta _1 \dot{R}}{2R^2}\,\delta + \frac{\beta _2 \delta \tau }{2R} + O(\delta ^3) \biggr ). \end{aligned} \end{aligned}$$

The Wasserstein distance upper bound is then computed by integration with respect to $\mu _{x_0}^{\sigma ,\varepsilon }$ using the coordinate representation of Remark 3.9 asapplying on the last line that $\Vert x_0-y\Vert = \delta \bigl ( 1 - \frac{\delta ^2}{24R^2}\bigr ) +O(\delta ^4)$. In the integral on the first line, terms of odd order vanish upon integration, and the remaining terms amount to integration of quadratic polynomials.

We now address the lower bound. Analogously to the plane curve case, define the test function for the Kantorovich–Rubinstein duality as

$$\begin{aligned} f(\phi (\alpha ,\beta _1,\beta _2)) :=\biggl \langle \phi (\alpha ,\beta _1,\beta _2)- x_0, \frac{y-x_0}{\Vert y-x_0\Vert }\biggr \rangle \end{aligned}$$

which is again clearly 1-Lipschitz in $\mathbb {R}^3$. We wish to apply Lemma 2.25 to show the lower bound and upper bound coincide up to $O(\delta ^4)$. Noting that $y-x_0 = \phi (\delta ,0,0)-\phi (0,0,0)$, we deduce from (3.9) that

$$\begin{aligned} \begin{aligned} y-x_0&= \delta \biggl ( 1- \frac{\delta ^2}{6R^2} + O(\delta ^3) \biggr )\hspace{0.55542pt}\dot{\gamma }(0) + \delta \biggl (\frac{\delta }{2R} + O(\delta ^2) \biggr )\hspace{0.55542pt}\textbf{n}(0) + O(\delta ^3)\hspace{0.55542pt}\textbf{b}(0),\\ \Vert y-x_0\Vert&= \delta \biggl ( 1 - \frac{\delta ^2}{24R^2} + O(\delta ^3) \biggr ), \end{aligned} \end{aligned}$$

and so

$$\begin{aligned} \begin{aligned} \frac{y-x_0}{\Vert y-x_0\Vert }&= \biggl (1-\frac{\delta ^2}{8R^2} + O(\delta ^3) \biggr )\hspace{0.55542pt}\dot{\gamma }(0) + \biggl (\frac{\delta }{2R} + O(\delta ^2)\biggr )\hspace{0.55542pt}\textbf{n}(0) + O(\delta ^2)\hspace{0.55542pt}\textbf{b}(0). \end{aligned} \end{aligned}$$

Therefore

$$\begin{aligned} \begin{aligned}&f(\phi (T(\alpha ,\beta _1,\beta _2))) - f(\alpha ,\beta _1,\beta _2) \\&\;\;= \biggl \langle T(\phi (\alpha ,\beta _1,\beta _2)) -\phi (\alpha ,\beta _1,\beta _2), \frac{y-x_0}{\Vert y-x_0\Vert } \biggr \rangle \\&\;\;= (\delta -2\alpha ) \biggl ( 1 -\frac{\beta _1}{R} - \frac{\delta ^2}{24R^2} + \frac{\delta \alpha }{6R^2} - \frac{\alpha ^2}{6R^2} + \frac{\beta _1 \delta \dot{R}}{2R^2} + \frac{\beta _2 \delta \tau }{2R} + O(\delta ^3) \biggr ). \end{aligned} \end{aligned}$$

This is the same expression as for $\Vert T(\phi (\alpha , \beta _1,\beta _2))-\phi (\alpha ,\beta _1,\beta _2)\Vert$, hence Lemma 2.25 applies and the lower and upper bounds agree up to $O(\delta ^4)$. $\square$

### Surface

We now consider a smooth 2-surface $M \subset \mathbb {R}^3$ and $\gamma :(-1,1) \rightarrow M$ a unit speed geodesic in *M*, denoting again $x_0 :=\gamma (0), y:=\gamma (\delta )$ for $\delta >0$ sufficiently small. Let $\textbf{n} \in \Gamma (TM^\perp )$ be the unit normal vector field and $\textbf{m} \in \Gamma (TM|_\gamma )$ the unit vector field along $\gamma$ orthogonal to the velocity $\dot{\gamma }$. Both $\textbf{n}$ and $\textbf{m}$ are unique up to sign.

**Fig. 3:**
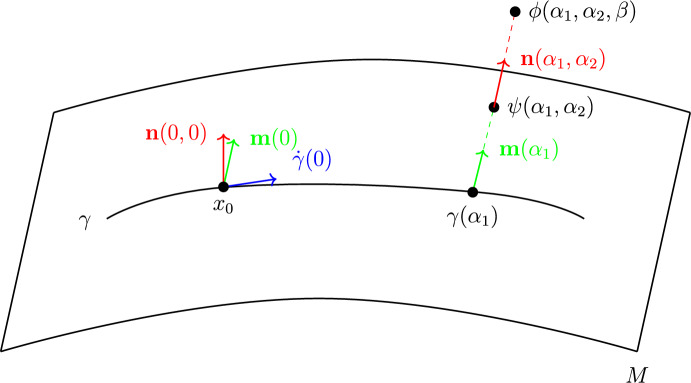
Fermi coordinates along $\gamma$ adapted to the surface *M* embedded in $\mathbb {R}^3$

#### Definition 3.11

- Define the Fermi coordinates $\psi :(-\delta _0,\delta _0) \hspace{1.111pt}{\times }\hspace{1.111pt}(-\varepsilon _0,\varepsilon _0) \rightarrow M$ along $\gamma$ in *M* as
  $$\begin{aligned} \psi (\alpha _1,\alpha _2) = \exp _{M,\gamma (\alpha _1)}(\alpha _2 \textbf{m}(\alpha _1)). \end{aligned}$$
- Define the Fermi coordinates $\phi :(-\delta _0,\delta _0) \hspace{1.111pt}{\times }\hspace{1.111pt}(-\varepsilon _0, \varepsilon _0) \hspace{1.111pt}{\times }\hspace{1.111pt}(-\sigma _0, \sigma _0) \rightarrow \mathbb {R}^3$ along $\gamma$ in $\mathbb {R}^3$ adapted to the surface *M* as
  $$\begin{aligned} \phi (\alpha _1,\alpha _2,\beta ) = \psi (\alpha _1,\alpha _2) + \beta \textbf{n}(\alpha _1,\alpha _2). \end{aligned}$$
  See Fig. 3 for a graphical representation of $\psi$ and $\phi$.
- For $i,j \in \{1,2\}$ denote the components of the second fundamental form in the Fermi coordinates

#### Remark 3.12

We point out that we overload the second fundamental form symbol  depending on the context of use. In the notation (1.1) and in the statements of Theorem 3.16 and Theorem 4.1, the subscript is the point $x_0$ on the manifold and the bracket arguments are tangent vectors. On the other hand, in coordinate computations taking place in the proofs, the subscripts will represent components with respect to the Fermi frame at Fermi coordinates $\alpha ,\beta$ in brackets.

Similarly to the Frenet–Serret frame in the case of a planar curve, we now consider the orthonormal frame $(\dot{\gamma }, \textbf{m},\textbf{n})$ with the intent to expand at $x_0$, i.e. $\alpha _1=\alpha _2=\beta =0$.

#### Lemma 3.13

The first derivatives of the normal vector field at $(\alpha _1,\alpha _2) =\textbf{0}$ areHence the derivatives of $\phi$ at $(\alpha _1,\alpha _2,\beta ) =\textbf{0}$ up to third order are

#### Proof

The derivatives involving $\partial _\beta$ are clear, recalling the definition

$$\begin{aligned} \phi (\alpha _1,\alpha _2,\beta ) :=\psi (\alpha _1,\alpha _2) + \beta \textbf{n}(\alpha _1,\alpha _2), \end{aligned}$$

and the first derivatives in $\alpha _1,\alpha _2$ follow from the definition of $\psi (\alpha _1,\alpha _2)$.

For $\partial _{\alpha _1} \textbf{n}(\textbf{0})$ we check its components with respect to the frame $(\dot{\gamma }, \textbf{m}, \textbf{n})$,and similarly for $\partial _{\alpha _2} \textbf{n}(\textbf{0})$.

For the second derivatives in $\alpha _1,\alpha _2$ at $(\alpha _1,0,0)$ for any $\alpha _1 \in (-\delta _0,\delta _0)$ and $j=1,2$,having introduced the term $\nabla ^M_{\partial _{\alpha _1}} \partial _{\alpha _j} \psi (\alpha _1,0)$, which vanishes for $j=1,2$, because $\alpha _1 \mapsto \psi (\alpha _1,0)$ is a geodesic on *M* and $\textbf{m}(\alpha _1)$ is the parallel translation of $\textbf{m}(0)$ along $\gamma$. By the same argument, for any $(\alpha _1, \alpha _2) \in (-\delta _0,\delta _0) \hspace{1.111pt}{\times }\hspace{1.111pt}(-\varepsilon _0,\varepsilon _0)$,because $\alpha _2 \mapsto \psi (\alpha _1,\alpha _2)$ is a geodesic for every $\alpha _1 \in (-\delta _0,\delta _0)$.

For the second derivatives in $\beta$ and one of $\alpha _1$ and $\alpha _2$, deduce $\partial _\beta \partial _{\alpha _i} \phi (\textbf{0}) = \partial _{\alpha _i} \textbf{n}(\textbf{0})$ and plug in for $\partial _{\alpha _i} \textbf{n}(\textbf{0})$.

For the third derivatives at $(\alpha _1,\alpha _2,\beta ) =\textbf{0}$, write by the chain ruleand plug in for $\partial _{\alpha _i} \textbf{n}(\textbf{0})$ in each. $\square$

Denote $D_u V$ the plain derivative in the direction $u \in \mathbb {R}^n$ of a vector field *V* as a smooth map from an open subset of $\mathbb {R}^n$ to $\mathbb {R}^n$.

#### Notation 3.14

Denote $$\widetilde{B}_{\sigma ,\varepsilon } :=\{(\alpha _1,\alpha _2,\beta )\,{:}\, \alpha _1^2+\alpha _2^2< \varepsilon ^2, |\beta | < \sigma \} \subset \mathbb {R}^3$$.

Consider the family of surfaces $\{ \phi (U, \beta ) \,{:}\, \beta \in (-\sigma _0, \sigma _0) \}.$ For any $\beta \in (-\sigma _0,\sigma _0)$, we denote the unit normal vector field of the surface as $\textbf{n}(\alpha _1,\alpha _2)$, which is unique up to sign. The corresponding mean curvature is:

$$\begin{aligned} H(\phi (\alpha _1,\alpha _2,\beta )) = \biggl \langle \textbf{n}(\alpha _1,\alpha _2), \sum _{i=1}^2 \nabla ^{\mathbb {R}^3}_{e_i} e_i(\phi (\alpha _1,\alpha _2,\beta )) \biggr \rangle \end{aligned}$$

where $(e_1,e_2)$ is an orthonormal frame on each $\phi (U,\beta )$.

#### Remark 3.15

**Fig. 4:**
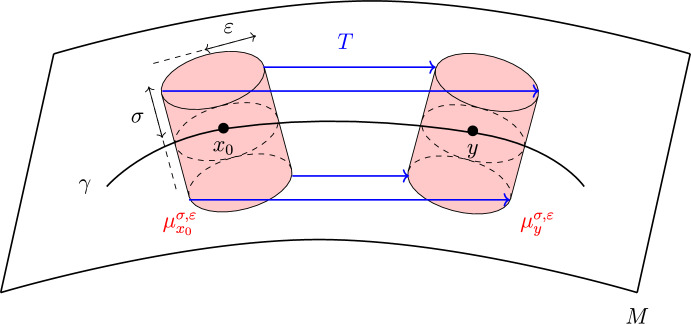
Test measures in red with some transport pairs of *T* in blue (Color figure online)

**Fig. 5:**
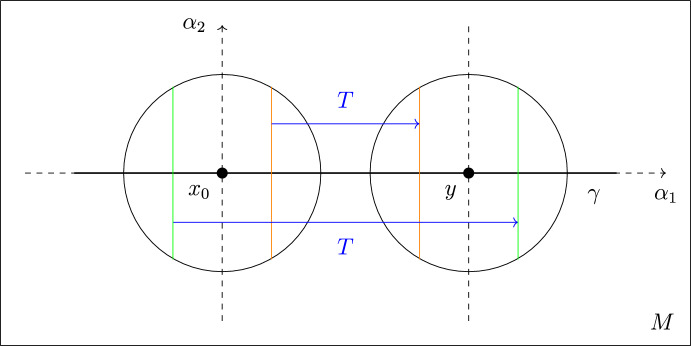
Top-down perspective for the transport map *T*

**Fig. 6:**
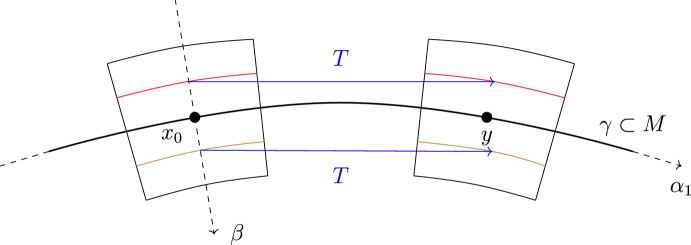
Cross-sectional perspective for the transport map *T*

1. As a special case of Lemma 2.17, the test measures at $y=\gamma (\delta )$ in these Fermi coordinates are 3.10 where $r(\alpha ,\beta )=O(\delta ^2)$ is a second order remainder.
2. The proposed transport map of Definition 2.18 reduces, in this case, to
  $$\begin{aligned} \begin{aligned} T(\phi (\alpha _1,\alpha _2,\beta ))&= \phi \bigl (\delta -\alpha _1, \alpha _2 + O(\delta ^3), \beta +O(\delta ^3)\bigr ). \end{aligned} \end{aligned}$$
  See Figs. 4, 5 and 6 for a pictorial representation of this map.

#### Theorem 3.16

Let *M* be an isometrically embedded surface in $\mathbb {R}^3$, let $x_0$ be a point and $(e_1,e_2)$ an orthonormal basis of principal curvature directions at $x_0$. Let $\gamma$ be a unit speed geodesic in *M* with $\gamma (0)=x_0$, $\dot{\gamma }(0)=e_1$ and denote $y=\gamma (\delta )$. For all $\delta , \varepsilon , \sigma >0$ sufficiently small with $\sigma \vee \varepsilon \leqslant {\delta }/{4}$, it holds that

#### Remark 3.17

If we set $\varepsilon =\frac{2\sqrt{2}}{\sqrt{3}}\sigma$, we note that the bracket on the right reduces to 1. This is due to the effects of second fundamental form and the curvature of the submanifold cancelling out, so it would appear in such special case that the coarse extrinsic curvature is flat, even though the second fundamental form may be non-vanishing. Such a special case is due to having an additional degree of freedom because of the additional $\sigma$ parameter and the sign of the $\sigma ^2$ term happens to oppose that of the $\varepsilon ^2$ term. The extrinsic curvature should thus be seen as encapsulated by varying both $\sigma$ and $\varepsilon$ in $W_1(\mu _{x_0}^{\sigma ,\varepsilon }, \mu _y^{\sigma ,\varepsilon })$.

#### Proof

The conclusion of Corollary 2.13 holds, so we may compute $W_1(\mu _{x_0}^{\sigma ,\varepsilon }, T_* \mu _{x_0}^{\sigma ,\varepsilon })$ instead. For every point $\phi (\alpha _1,\alpha _2,\beta )$, expanding up to third order and using the list of derivatives of Lemma 3.13, we collect terms as components of the frame $(\dot{\gamma }, \textbf{m},\textbf{n})$ at $\textbf{0}$,3.11While the terms $\beta \alpha _i \alpha _j \partial _\beta \partial _{\alpha _i} \partial _{\alpha _j} \phi (\textbf{0})$ are only of order 3, they are linear in $\beta$, and hence will not influence the integral with respect to $\mu _{x_0}^{\sigma , \varepsilon }$ up to $O(\delta ^4)$. In the same way an expression for the proposed transport

$$\begin{aligned} T(\phi (\alpha _1,\alpha _2,\beta )) = \phi \bigl (\delta -\alpha _1,\alpha _2+O(\delta ^3),\beta +O(\delta ^3)\bigr ) \end{aligned}$$

can be obtained by making corresponding substitutions for the components in the above expression for $\phi (\alpha _1,\alpha _2,\beta )$. Then the pointwise transport vector is3.12and its magnitude is3.13Using the density of the test measure $\mu _{x_0}^{\sigma ,\varepsilon }$ in Fermi coordinates given by (3.10), the upper bound isIn the third equality, we plugged in for $\Vert T(\phi (\alpha ,\beta )) -\phi (\alpha ,\beta )\Vert$ as computed above and used that terms of odd order in one of $\alpha _1,\alpha _2,\beta$ integrate to 0 and again absorbed higher order terms into $O(\delta ^4)$. On the last line, we used thatWe proceed with showing the lower bound. Define

$$\begin{aligned} p(\alpha _2,\beta ) :=\frac{\phi (\delta ,\alpha _2,\beta )-\phi (0,\alpha _2,\beta )}{\Vert \phi (\delta ,\alpha _2,\beta )-\phi (0,\alpha _2,\beta )\Vert } \end{aligned}$$

and the test function

3.14 $$\begin{aligned} f(\phi (\alpha _1,\alpha _2,\beta )) :=\langle \phi (\alpha _1,\alpha _2,\beta ) - x_0, p(\alpha _2,\beta )\rangle \end{aligned}$$

for the Kantorovich–Rubinstein duality, with the intention of applying Lemma 2.25 to conclude. We first expandDeduceand therefore3.15Then it can be verified using expansions (3.12) and (3.15) to compute the inner product that

$$\begin{aligned} \begin{aligned} f(Tz)-f(z)&= \bigl \langle T(\phi (\alpha _1,\alpha _2,\beta ))-\phi (\alpha _1,\alpha _2,\beta ),p(\alpha _2,\beta )\bigr \rangle \\&= \Vert T(\phi (\alpha _1,\alpha _2,\beta )) -\phi (\alpha _1,\alpha _2,\beta )\Vert + O(\delta ^4) \end{aligned} \end{aligned}$$

by comparison with (3.13).

It remains to show that the magnitude of the gradient of *f* satisfies

$$\begin{aligned} \sup _{z \in B_{2\delta }(x_0)}\Vert \nabla f(z)\Vert = 1 + O(\delta ^3). \end{aligned}$$

For this we need to expand the inverse matrix of the metric in Fermi coordinates. Using the expansion (3.11), computeWe shall label the term

$$\begin{aligned} r(\alpha ) :=\frac{1}{3} \,\alpha _1 \langle \partial _{\alpha _1}^2 \textbf{n}(\textbf{0}), \dot{\gamma }(0)\rangle + \frac{1}{3}\, \alpha _2 \langle \partial _{\alpha _1} \partial _{\alpha _2} \textbf{n}(\textbf{0}), \dot{\gamma }(0)\rangle . \end{aligned}$$

Then the metric matrix has the shape

$$\begin{aligned} G = \begin{pmatrix} g_{11} &  g_{12} &  0 \\ g_{21} &  g_{22} &  0 \\ 0 &  0 &  1 \end{pmatrix} \end{aligned}$$

withNote that the matrix is of the form

$$\begin{aligned} G = I + A \end{aligned}$$

with $A=O(\delta )$, which means the expansion of its inverse is

$$\begin{aligned} G^{-1} = I-A +A^2 + O(\delta ^3). \end{aligned}$$

We computeand thusFrom (3.15) we deduce the derivatives of the projection vector field in coordinates are

$$\begin{aligned} \begin{aligned} \partial _{\alpha _2} p(\alpha _2,\beta )&= O(\delta ^2)\hspace{0.55542pt}\dot{\gamma }(0) +O(\delta ) \hspace{0.55542pt}\textbf{m}(0) + O(\delta ) \hspace{0.55542pt}\textbf{n}(0),\\ \partial _\beta p(\alpha _2,\beta )&= O(\delta ^2)\hspace{0.55542pt}\dot{\gamma }(0) + O(\delta )\hspace{0.55542pt}\textbf{m}(0) +O(\delta ) \hspace{0.55542pt}\textbf{n}(0). \end{aligned} \end{aligned}$$

Then the first derivatives of the test function defined in (3.14) areThen the magnitude of the gradient is

$$\begin{aligned} \begin{aligned} \Vert \nabla f(\phi (\alpha _1,\alpha _2,\beta ) \Vert ^2&= (g^{11}\hspace{0.55542pt}{\circ }\hspace{1.111pt}\phi )(\partial _{\alpha _1}(f\hspace{0.55542pt}{\circ }\hspace{1.111pt}\phi ))^2 + 2 (g^{12}\hspace{0.55542pt}{\circ }\hspace{1.111pt}\phi )\hspace{0.55542pt}\partial _{\alpha _1}(f\hspace{0.55542pt}{\circ }\hspace{1.111pt}\phi )\hspace{0.55542pt}\partial _{\alpha _2} (f \hspace{0.55542pt}{\circ }\hspace{1.111pt}\phi ) \\&\qquad \qquad + (g^{22}\hspace{0.55542pt}{\circ }\hspace{1.111pt}\phi )(\partial _{\alpha _2}(f\hspace{0.55542pt}{\circ }\hspace{1.111pt}\phi ))^2+ (\partial _\beta (f \hspace{0.55542pt}{\circ }\hspace{1.111pt}\phi ))^2, \end{aligned} \end{aligned}$$

and we find the individual summandswhich indeed gives

$$\begin{aligned} \Vert \nabla f(\phi (\alpha _1,\alpha _2,\beta ) \Vert = 1+O(\delta ^3) \end{aligned}$$

as the first and second order terms cancel out. Hence Lemma 2.25 applies and we conclude the lower bound coincides up to $O(\delta ^4)$ with the upper bound. $\square$

## General Riemannian submanifolds

We now consider a Riemannian submanifold *M* of arbitrary dimension *m* and codimension *k* embedded isometrically in $\mathbb {R}^{m+k}$. Theorems 3.10 and 3.16 are thus special cases of Theorem 4.1 below. We begin by defining an orthonormal frame of $\mathbb {R}^{m+k}$-valued vector fields on a sufficiently small open domain *U* in the submanifold *M*, which is used to define the Fermi coordinates on *U* in this general setting.

### Frame extension

We take the ambient manifold to be $\mathbb {R}^{m+k}$. Recall the second fundamental form at a point $x \in M$ iswhere *W* is an arbitrary local vector field on *M* with $W(x) = w_2$. The mean curvature at *x* isfor an arbitrary orthonormal basis $(e_j)_{j=1}^m$ of $T_xM$. Both  and *H*(*x*) are normal to the submanifold, i.e.Recall from Definition 2.14 that the Fermi coordinates in *M* along $\gamma$ are given by

$$\begin{aligned} \psi (\alpha ) = \exp _{M, \gamma (\alpha _1)}\biggl ( \sum _{j=2}^m \alpha _j e_j(\alpha _1)\biggr ), \end{aligned}$$

where $(e_j(\alpha _1)_{j=1}^m$ is the parallel transport along $\gamma$ of an orthonormal basis $(e_j(0))_{j=1}^m$ of $T_{x_0}M$ with $e_1(0)=\dot{\gamma }(0)$. We refer back to Sect. 2 for properties of the Fermi chart.

Denote $\hat{\alpha } = (\alpha _2,\ldots ,\alpha _m)$ so that $\alpha =(\alpha _1,\hat{\alpha })$. Extend the frame $(e_j(\alpha _1))_{j=1}^m$ defined along $\alpha _1 \mapsto \gamma (\alpha _1)$ to $U \subset M$ by imposing

$$\begin{aligned} \frac{D}{ds}\, e_j(\alpha _1, s\hat{\alpha }) = 0, \end{aligned}$$

i.e. by parallel translating in *M* along the geodesic $s \mapsto \psi (\alpha _1, s\hat{\alpha })$.

Given an initial orthonormal basis $(\textbf{n}_i)_{i=1}^k$ of $T_{x_0}M^\perp$, first extend it to a frame along $\gamma$ by requiring that

$$\begin{aligned} \partial _{\alpha _1} \langle \textbf{n}_i(\alpha _1), e_j(\alpha _1)\rangle = 0 \;\; \text {and} \;\; (\partial _{\alpha _1} \textbf{n}_i(\alpha _1) )^\perp = 0 \quad \text {for all}\;\; \alpha _1 \in (-\delta _0,\delta _0). \end{aligned}$$

The first requirement implieswhich together with the second requirement implies the first order ODE4.1The solution exists and is unique by standard ODE theory. Having defined the frame $(\textbf{n}_i(\alpha _1))_{i=1}^k$ along the geodesic $\alpha _1 \mapsto \gamma (\alpha _1)$, we may also extend it to the submanifold by requiring that for every $\alpha _1 \in (-\delta _0,\delta _0)$ and $\hat{\alpha } \in \tilde{B}^{m-1}_{\varepsilon _0}$,Similarly to the above, the first requirement implies that for all $j =1,\ldots ,m$,

$$\begin{aligned} \begin{aligned} \biggl \langle \frac{d}{ds} \,\textbf{n}_i(\alpha _1, s\hat{\alpha }), e_j(\alpha _1, s\hat{\alpha })\biggr \rangle&=  -\biggl \langle \textbf{n}_i(\alpha _1, s\hat{\alpha }), \frac{d}{ds}\, e_j(\alpha _1, s\hat{\alpha }) \biggr \rangle , \end{aligned} \end{aligned}$$

and from the second requirement we conclude the frame satisfies the first order ODE

$$\begin{aligned} \frac{d}{ds}\, \textbf{n}_i(\alpha _1, s\hat{\alpha }) = - \sum _{j=1}^m\, \biggl \langle \textbf{n}_i(\alpha _1, s\hat{\alpha }), \frac{d}{ds}\, e_j(\alpha _1, s\hat{\alpha }) \biggr \rangle \hspace{1.111pt}e_j(\alpha _1,s\hat{\alpha }) \end{aligned}$$

along each geodesic $s \mapsto \phi (\alpha _1, s\hat{\alpha })$ in *M*.

With these concrete vector fields, recall the Fermi coordinates in $\mathbb {R}^{m+k}$ along $\gamma$ adapted to the submanifold *M* were defined in Definition 2.14 as

$$\begin{aligned} \phi (\alpha ,\beta ) = \psi (\alpha ) + \sum _{i=1}^k \beta _i \textbf{n}_i(\alpha ) \end{aligned}$$

and note that $\phi (\alpha ,\textbf{0}) = \psi (\alpha )$.

For every $\alpha _1 \in (-\delta _0,\delta _0)$, the map $\psi (\alpha _1, \hspace{0.55542pt}{\cdot }\hspace{1.111pt}):\widetilde{B}^{m-1}_{\varepsilon _0} \rightarrow M$ is the exponential chart on its image. It is known that the Christoffel symbols vanish at the centre for such charts, i.e.

$$\begin{aligned} \nabla ^M_{\partial _{\alpha _i} \psi } \partial _{\alpha _j}\psi (\alpha _1,\textbf{0}) = 0 \quad \text {for all}\;\; i,j=2,\ldots ,m. \end{aligned}$$

Moreover, since $\partial _{\alpha _j} \psi (\alpha _1,0) = e_j(\alpha _1)$ for $j=1,\ldots ,m$ is parallel transport of $e_j(0)$ along $\gamma$, also

$$\begin{aligned} \nabla ^M_{\partial _{\alpha _1}\psi } \partial _{\alpha _j} \psi (\alpha _1,\textbf{0}) = 0 \quad \text {for all}\;\; j=1,\ldots ,m, \end{aligned}$$

noting that $$\dot{\gamma }(\alpha _1) = \partial _{\alpha _1} \psi (\alpha _1,\textbf{0})$$.

Denote the components of the second fundamental form with respect to the Fermi coordinates asNote that the first index represents the normal direction and the latter two represent manifold directions. Then we can write for every $j,\ell =1,\ldots ,m$,4.2In addition, (4.1) can be written asThus the third derivatives with at least one in $\alpha _1$ are4.3

### Main theorem

In the statement of the theorem,  is the vector of second fundamental form. In the proof exclusively,  denotes the *ij*-component of the second fundamental form with respect to the Fermi frame at Fermi coordinates $\alpha ,\beta$.

#### [Style2 Style3 Style3]Theorem 4.1

Let *M* be an isometrically embedded submanifold of $\mathbb {R}^{m+k}$, and $\gamma$ a unit speed geodesic in *M* such that $\gamma (0) = x_0$ and $\gamma (\delta )=y$. Let $(e_j)_{j=1}^m$ be an orthonormal basis of $T_{x_0}M$ with $e_1 = \dot{\gamma }(0)$ and assume that  for all $j=2,\ldots ,m$. Then for every $\sigma ,\varepsilon , \delta >0$ sufficiently small with $\sigma \vee \varepsilon \leqslant {\delta }/{4}$ it holds that

#### Remark 4.2

We point out two special cases:

- If the submanifold has dimension 1 then the condition on the second fundamental form is trivially satisfied as there are no submanifold directions other than that of the curve itself. In this case  and hence . This is the square curvature of the curve and for $m=1$, $k=2$ agrees with Theorem 3.10.
- If the submanifold has codimension 1 with a normal vector field $\textbf{n}$ on the submanifold, then the orthonormal eigenbasis of  satisfies the condition  for $i\ne j$. Such a basis always exists as  is symmetric and consists of the so-called principal curvature directions. Thus for $m=2$, $k=1$, we obtain Theorem 3.16 as a special case.
- In general codimension, however, such a basis may not exist for a general submanifold, hence the assumption on the second fundamental form needs to be made and is highly restrictive. If this assumption was dropped, the upper bound for the Wasserstein distance via the proposed transport map would still apply. However, the computation of the lower bound using a projection plane, as done in the proof of Theorem 3.16 and applied again in the proof below, would yield additional lower order terms not agreeing with the upper bound. This is symptomatic of the non-optimality of the transport map up to third order. The more general computation including the off-diagonal terms to show this is straightforward but rather lengthy and is thus omitted. Qualitatively, the issue is that the off-diagonal terms of the second fundamental form introduce a deformation of the supports of the test measures which is not easily remedied and leaves the fully general case open. The deformation arises because the principal curvature directions above the reference point $x_0$ for each leaf of the foliation of the tubular neighbourhood change their vertical alignment as we consider leaves further away from the base submanifold *M*. On the other hand, the diagonal assumption on the second fundamental form ensures an aligned stacking of principal curvature directions of leaves above $x_0$, leading to the favourable cylinder-like support of the test measures.
- For the interpretation of the special case of the parameters $\varepsilon =\sqrt{\frac{2(m+2)}{k+2}}\sigma$, we refer back to Remark 3.17.

#### Proof of Theorem 4.1

Expand the Fermi chart up to and including third order asFrom the definition of the Fermi chart and (4.2), (4.3), we have the derivatives at the origin on the right-hand side:With these we obtain:In the above, the sum of third derivative terms in $\alpha$ was split into those that involve at least one power in $\alpha _1$, for which we have a formula, and those that don’t. The other third derivatives $\partial _{\alpha _i} \partial _{\alpha _r} \partial _{\alpha _\ell } \phi (\textbf{0})$ for $i,r,\ell \geqslant 2$ are not easily written in Fermi coordinates, but will not be needed for our computations. Rearranging the terms, we write $\phi$ in terms of the basis $(e_1(0),\ldots ,e_m(0)$, $\textbf{n}_1(\textbf{0}),\ldots , \textbf{n}_k(\textbf{0}))$ and apply the assumption :4.4We will henceforth denote

$$\begin{aligned} r_i(\alpha ):=\frac{1}{2}\, \alpha _1 \langle \partial _{\alpha _1}^2 \textbf{n}_i(\textbf{0}), e_1(0)\rangle + \sum _{\ell =2}^m \alpha _\ell \langle \partial _{\alpha _\ell } \partial _{\alpha _1} \textbf{n}_i(\textbf{0}),e_1(0) \rangle . \end{aligned}$$

Let *T* be the transport map defined in Definition 2.18. With asymptotic notation for the third order terms,

$$\begin{aligned} T(\phi (\alpha _1,\hat{\alpha },\beta )) = \phi (\delta -\alpha _1,\hat{\alpha }, \beta +O(\delta ^3)). \end{aligned}$$

In the expansion of $\phi$ above, from the third derivatives in $\alpha$ we only needed to specify those involving $\alpha _1$, because the transport map *T* changes only the first coordinate up to $O(\delta ^3)$. These derivatives were given by (4.3). Then the pointwise transport vector is4.5Therefore, using the expansion $\sqrt{1+x}=1+\frac{1}{2}x-\frac{1}{8}x^2 + O(x^3)$, the pointwise transport distance is4.6Lemma 2.17 expressed the density of the test measure $\mu _{x_0}^{\sigma ,\varepsilon }$ in Fermi coordinates up to second order. Denoting the second order remainder of the density as $r(\alpha ,\beta )$, the density simplifies to give4.7where the form of the normalizing factor in the denominator is deduced from the two factsWe deduce the upper bound in the statement of Theorem 4.1 by computing the integral on the right side of the inequalityup to and including third order terms. Using the product of expressions (4.7) and (4.6), this amounts to integrating a quadratic polynomial in $\alpha ,\beta$. First, as terms with odd power in one of the coordinates vanish, we simplify the integral toWe now use the fact that the average integral of the square of any coordinate over a *d*-dimensional ball of arbitrary radius $r >0$ iswhere  denotes the integral normalised by the volume of the ball and using that

$$\begin{aligned} |B_r^d| = \frac{\pi ^{{d}/{2}}}{\Gamma ({d}/{2}+1)} \, r^{d}, \quad |\partial B_{s}^{d}|=\frac{2 \pi ^{{d}/{2}}}{\Gamma ({d}/{2})}\, s^{d-1}. \end{aligned}$$

This in particular givesThenFurthermore, from (4.6) for $\alpha = 0, \beta =0$ we deduceTherefore, we can rewrite in terms of the Euclidean distance:We now address the lower bound. Denoting

$$\begin{aligned} p(\hat{\alpha },\beta ) :=\frac{\phi (\delta ,\hat{\alpha },\beta )-\phi (0,\hat{\alpha },\beta )}{\Vert \phi (\delta ,\hat{\alpha },\beta )-\phi (0,\hat{\alpha },\beta )\Vert }, \end{aligned}$$

emphasizing that this vector does not depend on $\alpha _1$, we propose

$$\begin{aligned} \begin{aligned} f(\phi (\alpha , \beta )) :=\langle \phi (\alpha ,\beta )- x_0, p(\hat{\alpha },\beta ) \rangle \end{aligned} \end{aligned}$$

as the test function for Kantorovich–Rubinstein duality, with the intention of applying Lemma 2.25 to conclude the upper bound is also a lower bound up to $O(\delta ^4)$. We deduce from (4.5) that4.8Then it can be verified, using the expansions (4.5) and (4.8) to compute the inner product up to and including third order terms, that

$$\begin{aligned} \begin{aligned} f(T(\phi (\alpha ,\beta ))) - f(\phi (\alpha ,\beta ))&= \bigl \langle T(\phi (\alpha ,\beta )) - \phi (\alpha ,\beta ) + O(\delta ^4), p(\hat{\alpha },\beta )\bigr \rangle \\&= \Vert T(\phi (\alpha ,\beta )) - \phi (\alpha ,\beta )\Vert + O(\delta ^4) \end{aligned} \end{aligned}$$

by comparison with (4.6).

Finally, we wish to compute the square magnitude of the gradient of the test function in order to verify that its supremum over $B_{2\delta }(x_0)$ is $1+O(\delta ^3)$ for Lemma 2.25 to apply. For this we need to establish the Riemannian metric in Fermi coordinates $g_{ij} = \langle \partial _{\alpha _i}\phi , \partial _{\alpha _j} \phi \rangle .$ The first derivatives of the Fermi chart are deduced by differentiating (4.4) asThen the entries of the inverse metric matrix are computed from these to beNote that $\partial _{\alpha _1}\phi$ and $g_{11}$ needed to be expanded up to second order due to the particular role of the first coordinate. For the rest, expansion up to first order is sufficient. The above means the metric matrix has the block structure

$$\begin{aligned} G = \begin{pmatrix} (g_{j \ell })_{j,\ell \leqslant m} &  O(\delta ^2) \\ O(\delta ^2) &  I_k \end{pmatrix}. \end{aligned}$$

In particular, denotingand the matrixhaving used that $a_{1j} =0$ for $j=2,\ldots ,m$ as  by assumption, we can write

$$\begin{aligned} G= I_{m+k}+ \begin{pmatrix}A &  O(\delta ^2) \\ O(\delta ^2) &  \textbf{0} \end{pmatrix}. \end{aligned}$$

Noting that the second matrix is $O(\delta )$, the expansion of its inverse is

$$\begin{aligned} G^{-1} = \begin{pmatrix} I_m - A + A^2 + O(\delta ^3) &  O(\delta ^2) \\ O(\delta ^2) &  I_k + O(\delta ^4) \end{pmatrix} \end{aligned}$$

due to the block structure. Computingwe deduceby plugging in for $a_{j \ell }$ and *b*, and alsoWe remark that for $j, \ell \geqslant 2$ the expansion of $g^{j\ell }$ up to the linear term suffices for the computations to follow, while the expansion of $g^{11}$ up to second order is necessary.

We now compute the expansions of the derivatives of the test function. The first derivatives of the projection vector field in coordinates can be computed from (4.8) as

$$\begin{aligned} \begin{aligned} \partial _{\alpha _j} p(\hat{\alpha },\beta )&= O(\delta ) \quad \text {for all}\;\; 2 \leqslant j \leqslant m, \\ \partial _{\beta _i} p(\hat{\alpha }, \beta )&= O(\delta ) \quad \text {for all}\;\; 1 \leqslant i \leqslant k. \end{aligned} \end{aligned}$$

Then computing the inner products, using (4.4) for the derivatives of the chart,and for $2 \leqslant j \leqslant m$,

$$\begin{aligned} \begin{aligned} \partial _{\alpha _j} (f \hspace{0.55542pt}{\circ }\hspace{1.111pt}\phi )(\alpha ,\beta ) = \langle \partial _{\alpha _j} \phi (\alpha ,\beta ), p(\hat{\alpha },\beta ) \rangle + \langle \phi (\alpha ,\beta ) - x_0, \partial _{\alpha _j} p(\hat{\alpha },\beta )\rangle = O(\delta ^2), \end{aligned} \end{aligned}$$

and for $i \leqslant k$,We wish to compute

$$\begin{aligned} \begin{aligned} \Vert \nabla f(\phi (\alpha ,\beta ))\Vert ^2&= \sum _{j,\ell =1}^m g^{j\ell }(\phi (\alpha ,\beta ))\hspace{0.55542pt}\partial _{\alpha _j}(f \hspace{0.55542pt}{\circ }\hspace{1.111pt}\phi )(\alpha ,\beta )\hspace{0.55542pt}\partial _{\alpha _\ell } (f \hspace{0.55542pt}{\circ }\hspace{1.111pt}\phi )(\alpha ,\beta )\\&\quad + 2 \sum _{i=1}^{k} \sum _{j=1}^m g^{m+i,j}(\phi (\alpha ,\beta ))\hspace{0.55542pt}\partial _{\beta _i} (f \hspace{0.55542pt}{\circ }\hspace{1.111pt}\phi )(\alpha ,\beta )\hspace{0.55542pt}\partial _{\alpha _j} (f \hspace{0.55542pt}{\circ }\hspace{1.111pt}\phi )(\alpha ,\beta ) \\&\quad + \sum _{i=1}^{k}\, (\partial _{\beta _i} (f\hspace{0.55542pt}{ \circ }\hspace{1.111pt}\phi )(\alpha ,\beta ))^2. \end{aligned} \end{aligned}$$

The individual summands areAll first and second order terms vanish upon summation, hence we may conclude that $\Vert \nabla f(\phi (\alpha ,\beta ))\Vert ^2 = 1 + O(\delta ^3)$ as required. $\square$

## Applications

### Poisson point processes on manifolds

In applications one may wish to recover curvature information from coarse curvature of a random point cloud represented by a Poisson point process. Such an approach has already been investigated in [18] and [2] for the Ricci curvature and generalised Ricci curvature, respectively.

We first recall the definition of a Poisson point process. Let  be a $\sigma$-finite measure space,  the set of measures on  and  a probability space.

#### Definition 5.1

A Poisson point process on  with intensity measure $\mu$ is a random measure  (equivalently ) such that the following three properties hold:

- For all $\mu$-finite measurable sets :  is a $\text {Poisson}\hspace{0.55542pt}(\mu (A))$ random variable.
- For all disjoint, measurable $\mu$-finite sets :  and  are independent random variables.
- For all $\omega \in \Omega$:  is a measure on .

It turns out (see [20, Chapter 6]) that all Poisson point processes with a finite intensity measure take the form of a random empirical measure, i.e.where *N* is a  random variable, $(X_i)_{i\in \mathbb {N}}$ are independent $\mu$-distributed random variables on  and $(X_i)_{i\in \mathbb {N}}, N$ are independent. Denote the random set of points thus generated by  as

#### Notation 5.2

Let  be a sequence of Poisson point processes on the ambient space $\mathbb {R}^{m+k}$ with uniform intensity measure $n \textrm{vol}_{\mathbb {R}^{m+k}}(dz)$. Denote by  the discrete random set of points generated by . Let $x_0 \in M$, $(\delta _n)_{n \in \mathbb {N}}$, $(\sigma _n)_{n \in \mathbb {N}}, (\varepsilon _n)_{n \in \mathbb {N}}$ sequences of positive reals and $y_n :=\exp _{x_0}(\delta _n v)$ for a fixed unit vector $v \in T_{x_0}M$. As the discrete counterpart to the test measures $\mu _x^{\sigma ,\varepsilon }$, for any point $x \in M$ denote the random empirical measures adapted to the submanifold,If $\sigma _n \vee \varepsilon _n \leqslant {\delta _n}/{4}$ then $B_{\sigma _n,\varepsilon _n}(x_0) \cup B_{\sigma _n, \varepsilon _n}(y_n) \subset x_0 + [-2\delta _n,2\delta _n]^{m+k}$.

Using the following result proved in [18, Corollary 3], it is possible to quantify the approximation of the test measures by the empirical measures in the Wasserstein metric:

#### Lemma 5.3

For all $n \in \mathbb {N}$, it holds that5.1

We may then deduce that coarse curvature of point clouds with the empirical measures as test measures has the same limit as coarse extrinsic curvature if the intensity of the point process increases fast enough relative to the parameter $\delta _n$. DenoteThis leads immediately to a corollary of Theorem 4.1:

#### Proposition 5.4

Under the assumptions of Theorem 4.1, if the sequences $(\delta _n)_{n \in \mathbb {N}}$, $(\sigma _n)_{n \in \mathbb {N}}$ and $(\varepsilon _n)_{n \in \mathbb {N}}$ satisfy $\sigma _n \vee \varepsilon _n \leqslant {\delta _n}/{4}$ and $\log \hspace{0.55542pt}(n)\hspace{0.55542pt}n^{-\frac{1}{m+k}}=o(\delta _n^3)$, then

#### Proof

By the triangle inequality and (5.1),At the same time, from Theorem 4.1 we havewhich gives the final result upon substitution and taking the limit as $n \rightarrow \infty$. $\square$

### Retrieving mean curvature

Theorem 4.1 could in practice be exploited in the two settings already alluded to in the introduction, which considered the planar curve case for illustrative purposes. In the scope of generality of Theorem 4.1, we haveIn particular, we may distinguish two limit regimes:

- Assuming $\sigma = \Theta (\delta )$ and $\varepsilon = o(\sigma )$,  This represents a situation where one can obtain a sample from the ambient measure in a tubular neighbourhood of the surface. Decreasing $\varepsilon$ corresponds to localization of the geometric information thus retrieved.
- Assuming $\varepsilon = \Theta (\delta )$ and $\sigma =o(\varepsilon )$,  In this case, we have a noisy sample from the surface and obtain convergence of the coarse extrinsic curvature under attenuation of the noise as $\sigma$ decreases.

Note that these expressions depend on the vector *v* with $y_\delta = \exp _{M, x_0}(\delta v)$. We can remove this directionality by adding up coarse curvatures in all directions of an orthonormal frame at $x_0$, thus obtaining an expression involving the mean curvature.

Denote the square norm of the mean curvature vector as

$$\begin{aligned} \Vert H(x_0)\Vert ^2 = \sum _{i=1}^k \,\langle H(x_0), \textbf{n}_i(x_0)\rangle ^2 \end{aligned}$$

for an arbitrary orthonormal basis $(\textbf{n}_i(x_0))_{i=1}^k$ of the normal space .

#### Corollary 5.5

Let $(e_j)_{j=1}^m$ be an orthonormal basis of $\,T_{x_0}M$, and for $j=1, \dots , m$, let $y_j = \exp _{M, x_0}(\delta e_j)$. Assume that  for $i\ne j$. Then for all $\sigma ,\varepsilon ,\delta >0$ sufficiently small with $\sigma \vee \varepsilon \leqslant {\delta }/{4}$ it holds that

$$\begin{aligned} \begin{aligned} \sum _{j=1}^m \biggl ( 1- \frac{W_1(\mu _{x_0}^{\sigma ,\varepsilon }, \mu _{y_j}^{\sigma ,\varepsilon })}{\Vert x_0-y_j\Vert }\biggr )&= \biggl ( \frac{\varepsilon ^2}{2(m+2)} - \frac{\sigma ^2}{k+2} \biggr ) \Vert H(x_0)\Vert ^2 + O(\delta ^3). \end{aligned} \end{aligned}$$

#### Proof

We express the coarse curvatures using the expansion of Theorem 4.1 and sum up, noting that $j = 1,\ldots ,m$ indexing each direction plays the role of the first coordinate,completing the proof. $\square$

This implies that given the family of coarse curvatures

$$\begin{aligned} \biggl \{ \,1- \frac{W_1(\mu _{x_0}^{\sigma ,\varepsilon }, \mu _{y_j}^{\sigma ,\varepsilon })}{\Vert x_0-y_j\Vert }: \sigma , \varepsilon , \delta > 0, j = 1,\ldots , m \,\biggr \}, \end{aligned}$$

one can retrieve the square magnitude of the mean curvature vector of the surface at $x_0$ asIn conclusion, we introduced the notion of coarse extrinsic curvature of Riemannian submanifolds embedded isometrically in a Euclidean space and verified that in a scaled limit of the parameters we retrieve meaningful geometric information about the submanifold. As illustrative examples, in the case of a curve we retrieve the inverse squared radius of the osculating circle at a given point, while in the case of a 2-surface we obtain an expression in terms of the second fundamental form and mean curvature. Such coarse extrinsic curvatures can be combined to yield the square magnitude of the mean curvature as a scaled limit.
